# Mesoporous MOFs with ROS scavenging capacity for the alleviation of inflammation through inhibiting stimulator of interferon genes to promote diabetic wound healing

**DOI:** 10.1186/s12951-024-02423-6

**Published:** 2024-05-13

**Authors:** Fupeng Li, Zhiyuan Mao, Yun Du, Yuehan Cui, Shengbing Yang, Kai Huang, Jian Yang, Zhuoyuan Li, Yihao Liu, Jinlou Gu, Danru Wang, Chen Wang

**Affiliations:** 1 Department of Plastic and Reconstructive Surgery, Shanghai Ninth People’s Hospital, Shanghai Jiao Tong University School of Medicine, Shanghai, 200011 People’s Republic of China; 2 Shanghai Key Laboratory of Orthopaedic Implants, Department of Orthopaedic Surgery, Shanghai Ninth People’s Hospital, Shanghai Jiao Tong University School of Medicine, Shanghai, 200011 People’s Republic of China; 3 State Key Laboratory of Chemical Engineering, School of Chemical Engineering, East China University of Science and Technology, Shanghai, 200237 People’s Republic of China; 4 Key Laboratory for Ultrafine Materials of Ministry of Education, School of Materials Science and Engineering, East China University of Science and Technology, Shanghai, 200237 People’s Republic of China

**Keywords:** Nanozymes, Senescence, Ferroptosis, STING, Diabetic wound

## Abstract

Excessive production of reactive oxygen species (ROS) and inflammation are the key problems that impede diabetic wound healing. In particular, dressings with ROS scavenging capacity play a crucial role in the process of chronic wound healing. Herein, Zr-based large-pore mesoporous metal–organic frameworks (mesoMOFs) were successfully developed for the construction of spatially organized cascade bioreactors. Natural superoxide dismutase (SOD) and an artificial enzyme were spatially organized in these hierarchical mesoMOFs, forming a cascade antioxidant defense system, and presenting efficient intracellular and extracellular ROS scavenging performance. In vivo experiments demonstrated that the SOD@HMUiO-MnTCPP nanoparticles (S@M@H NPs) significantly accelerated diabetic wound healing. Transcriptomic and western blot results further indicated that the nanocomposite could inhibit fibroblast senescence and ferroptosis as well as the stimulator of interferon genes (STING) signaling pathway activation in macrophages mediated by mitochondrial oxidative stress through ROS elimination. Thus, the biomimetic multi-enzyme cascade catalytic system with spatial ordering demonstrated a high potential for diabetic wound healing, where senescence, ferroptosis, and STING signaling pathways may be potential targets.

**Graphical Abstract:**

**Figure:**
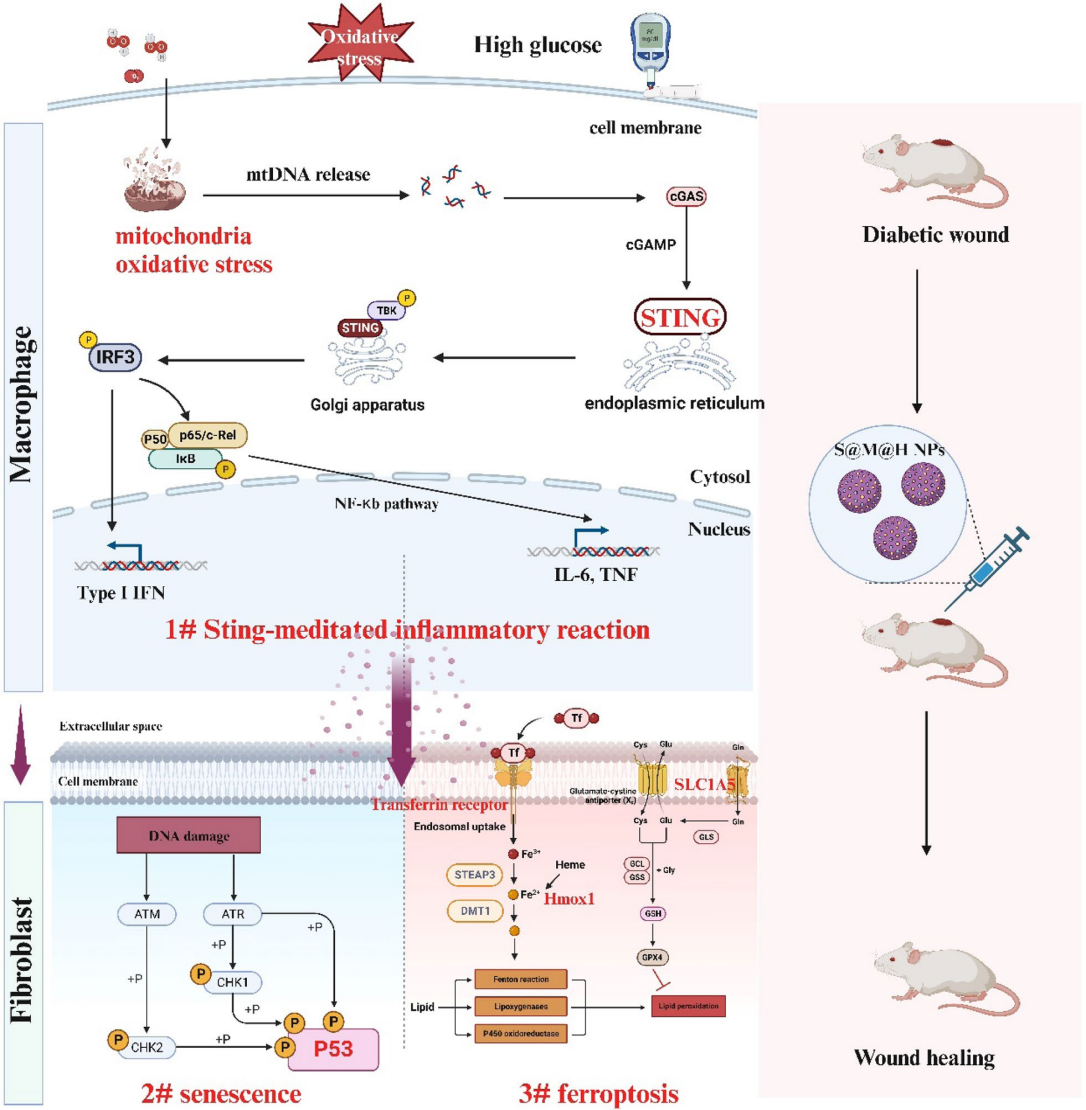
Schematic diagram showing the proposed mechanism that S@M@H NPs promoted diabetic wound healing

**Supplementary Information:**

The online version contains supplementary material available at 10.1186/s12951-024-02423-6.

## Introduction

Diabetes is a metabolic disease typically characterized by high blood sugar [1]. To date, more than 500 million people worldwide have diabetes. According to statistics, the incidence of diabetes among people aged 20–79 reached 10.5% in 2021 and is expected to rise to 12.2% in 2045 [2, 3]. Global health expenditure related to diabetes is estimated to be around $ 966 billion, creating a huge economic burden. Delayed wound healing is one of the most common complications of diabetes. More than 25% of patients with diabetes experience delayed wound healing. According to statistics, the 5-year mortality rate of patients with amputation, due to poor treatment of diabetic foot ulcers, is as high as 39–68% [4, 5]. Therefore, the promotion of diabetic wound healing is of great clinical significance as it is conducive to diabetic foot wound management, alleviates diabetic complications, and improves the quality of life of patients. Finding new therapy targets and developing innovative diabetic wound care treatments have the potential to generate significant economic benefits.

The oxidative stress microenvironment is a crucial factor that impairs wound healing [6, 7]. Reactive oxygen species (ROS), such as H_2_O_2_ and superoxide anion, are produced as metabolites under physiological conditions in vivo and play a key role in the regulation of cell functions. However, an overproduction of ROS will subject cells to oxidative stress, inducing a variety of deleterious cellular responses (e.g., apoptosis and necrosis), and triggering a myriad of pathologies, including atherosclerosis, neurodegeneration, inflammation, aging, hemochromatosis, and even cancer [8–10]. In diabetic wound healing, both local high glucose and chronic inflammation could lead to the overproduction of ROS, depletion of endogenous SOD enzymes, and redox imbalance [11–13]. Wound healing is a complex pathophysiological process including hemostatic, inflammatory, proliferating, and remodeling phases. After hemostasis phase, immune cells are recruited to the wound and secrete inflammatory factors to remove necrotic cells, extracellular matrix, and bacteria in the microenvironment, and stimulate angiogenesis simultaneously. Then, the inflammation gradually disappears, and the wound healing enters proliferating and remodeling phases. In the diabetic wound healing, the wound stagnates in the chronic inflammatory stage, which is not conducive to wound healing [14]. Overexpression of ROS activates inflammatory signaling pathways such as NF-κb, and enlarges inflammatory responses, which promotes matrix metalloproteinases production so that the wound healing process stays in its inflammatory stage [15]. Excessively produced matrix metalloproteinases degrade the extracellular matrix and hinder wound repair [2]. In general, oxidative stress and chronic inflammation constitute the microenvironment of diabetic wound and delay wound healing. However, the mechanism of oxidative stress to mediate chronic inflammation remains to be clarified. cGAS-STING is a crucial pathway responsible for the innate immune response. Recently, more and more researches have shown that the cGAS-STING pathway plays an important role in the progression of chronic inflammatory diseases [16–18]. This inspires us to explore whether chronic inflammation activated by oxidative stress is also through cGAS-STING pathway in diabetic wound.

Besides, the oxidatively stressed microenvironment impairs cells (e.g., fibroblasts, endothelial cells and macrophages) vitally for wound healing, resulting in collagen deposition disorders, angiogenesis abnormalities, as well as immune regulation disorders. In addition, the blocked microcirculation in patients with diabetes reduces oxygen supply, further hindering wound healing [19]. To rebuild the oxidatively stressed microenvironment of diabetic wound, the development of new treatments is in great need. However, degrading ROS under physiological conditions is a complex process and multiple enzymes are involved, such as SOD and catalase. The direct application of enzymes to eliminate ROS frequently faces significant hurdles: (1) enzymes are proteins which can be easily degraded by protease, making them difficult to store and transport. (2) The catalysis specificity of enzymes relies on their unique three-dimensional structures that are sensitive to the environment, including temperature and pH value [20]. Therefore, the development of orchestrated enzymes in artificial porous materials, such as polymersomes, liposomes, metal–organic particles, and mesoporous silica to avoid the mentioned limitations has aroused burgeoning interest.

In this study, we designed a catalase-mimicking Zr-based large-pore mesoporous metal–organic frameworks (mesoMOFs) to anchor the SOD and scavenge the excessive ROS via a cascade reaction. The integrated Mn-meso-tetra(4-carboxyphenyl) porphine (MnTCPP) in the framework of mesoMOFs served as catalase mimic while the inherent mesopores were available for the encapsulation and protection of SOD. Moreover, the mechanism by which the SOD@HMUiO-MnTCPP nanoparticles (S@M@H NPs) regulate wound healing under oxidative stress is also elucidated. H_2_O_2_ in diabetic wound microenvironment induces mitochondrial oxidative stress in macrophages, which activates cGAS-STING pathway, and finally leads to the secretion of inflammatory factors [21–23]. The ROS cascade elimination system of S@M@H NPs effectively reduced the mitochondrial oxidative stress-mediated cGAS-STING pathway activation in macrophages. In addition, STING-activated inflammatory macrophages further damaged fibroblasts responsible for wound repair, leading to ferroptosis and senescence phenotypes in fibroblasts. S@M@H NPs inhibit inflammatory macrophages activation and protect fibroblasts from senescence and ferroptosis in inflammatory microenvironment. Finally, we demonstrated that S@M@H NPs effectively promoted wound healing in diabetic mice in vivo.

## Experimental section

### Materials

Zirconium(IV) oxynitrate dihydrate (ZrO(NO_3_)_2_·2H_2_O) was purchased from Shanghai Macklin Biochemical Co., Ltd. Sodium perchlorate monohydrate (NaClO_4_·H_2_O) was purchased from Sinpharm Chemical Reagent Co., Ltd. Pluronic F127(PEO_106_PPO_70_PEO_106_) and 1,3,5-trimethylbenzene (TMBE) were supplied by Sigma-Aldrich. 2-Aminoterephthalic acid (BDC-NH_2_) was supplied by Alfa Aesar Chemicals. Mn-meso-tetra(4-carboxyphenyl) porphine (TCPP) was purchased from J&K Scientific Ltd., China. Acetic acid (AA) were purchased from Sinpharm Chemical Reagent Co., Ltd. Superoxide dismutase (SOD) was obtained from Shanghai Aladdin Biochemical Technology Co., Ltd. All reagents were of analytical grade and used without further purification.

### Synthesis of SOD@HMUiO-MnTCPP NPs

S@M@H NPs were synthesized according to a previously described method with hydrophobic TMBE to enlarge the mesopore size [24, 25].

### Characterization of SOD@MnTCPP@HMUIO

The powder X-ray diffraction (XRD) patterns were obtained using Cu Kα radiation. Transmission electron microscopy (TEM) was conducted on a JEM-2100F electron microscope. DLS was performed using a NICOMP Particle Sizing system. N_2_ sorption isotherms were recorded using a surface area and pore size analyzer. All of the samples were degassed under vacuum at 120 °C for 12 h prior to analysis. The specific surface area was calculated using the Brunauer–Emmett–Teller (BET) method using adsorption data at a relative pressure (P/P_0_) lower than 0.15. Confocal laser scanning microscope (CLSM) was conducted on a LEICA TCS SP8 instrument.

### O_2_^−^ scavenging activity of SOD@HMUiO-MnTCPP NPs

The O_2_^−^ scavenging activity was assessed using a superoxide anion assay kit according to the manufacturer’s instructions. Different concentrations of S@M@H NPs (0–1 µg mL^−1^) were added to the working solution. The absorbance was measured at 550 nm using a plate reader after standing for 10 min.

### CAT-like activity

The H_2_O_2_ scavenging capacity of the S@M@H NPs was tested using a H_2_O_2_ detection kit. H_2_O_2_ reacts with ammonium molybdate to form a stable yellow complex that displays an absorbance peak at 405 nm. Various concentrations of S@M@P NPs (25–200 µg mL^−1^) were incubated with 2 mM H_2_O_2_ at 37 °C for 2 h, respectively. After the reaction, the concentration of the remaining H_2_O_2_ was determined from the OD value of the solution at 405 nm. Hydroxyl radicals are produced via the Fenton reaction, in which H_2_O_2_ is catalyzed by ferrous ions.

### Cell culture

Three different cell models were used in vitro. The mouse fibroblast cell line (L929) is an internationally recognized cell line routinely used in in vitro cytotoxicity assessments. The other two cell models used were Raw 264.7 cells, mouse mononuclear macrophage leukemia cells; and HUVEC cells which are human umbilical vein endothelial cells. All cell culture media (DMEM medium) were supplemented with 10% fatal bovine serum (FBS) and 1% penicillin/streptomycin. The cells were cultured at 37 °C in a 5% CO_2_ incubator. The medium was changed every 2 days. L929 cells and HUVEC were routinely harvested using 0.25% trypsin solution before reaching 80% confluence, whereas Raw 264.7 cells were harvested using a cell scraper. Raw 264.7 cells were cultured under H_2_O_2_ (from 200 to 800 μM) and H_2_O_2_ + S@M@H NPs conditions. The medium was replaced with fresh complete medium after 24 h. After an additional 24 h incubation, the media was collected for later use. And the supernatants were mixed with fresh Dulbecco’s-modified-eagle-medium (DMEM) at a ratio of 1:1 and added to L929 cells for co-culture. This macrophage-derived conditioned medium named CM, marked as H_2_O_2_-stimulated Raw 264.7 cells (H_2_O_2_-CM) and H_2_O_2_ + S@M@H NPs-stimulated Raw 264.7 cells (S@M@H NPs-CM).

### Cytotoxicity assay

The cytotoxicity of S@M@P NPs was evaluated using the Cell Counting Kit 8 (CCK-8) assay. In brief, 100 μL of L929 cells, Raw 264.7 cells, and HUVEC cells in DMEM were placed into 96-well plates at a density of 8 × 10^3^ cells per well at 37 °C. After incubation overnight, the old culture medium was replaced with 100 μL fresh DMEM medium containing various concentrations of S@M@P NPs (0, 12.5, 25, 50, 100, 200, and 400 μg mL^−1^). After 24 h, the cells were lightly rinsed three times with sterile phosphate buffered saline (PBS). Then, 100 μL of fresh culture media (no serum) containing 10 μL of CCK-8 solution was added and incubated for 2 h at 37 °C. Subsequently, relative cell viability was calculated by measuring the absorbance of CCK-8 at 450 nm using a microplate reader.

### Protecting L929 cells from STING-activated macrophages

The cells were inoculated into 96-well plates (8 × 10^3^ cells/well). After incubation overnight, the old medium was discarded and the cells were exposed to H_2_O_2_-CM and S@M@H NPs-CM for another 24 h incubation. Finally, cell viability was determined using a CCK-8 assay. For the live/dead assay, the L929 cells were seeded in confocal dishes at a density of 1 × 10^5^ cells/well. After culturing for 24 h, the cells were stained with a working solution containing 2 μM calcein acetoxymethyl (calcein AM) and 4.5 μM propidium iodide for 15 min at 37 °C before observation via laser confocal microscopy. A wound-healing assay was performed to evaluate the migration of L929 cells. L929 cells were seeded in a six-well plate at a density of 2 × 10^5^ cells mL^−1^ until they reached 90% confluency. A straight, parallel, and cell free wound in each well was made by a 200 μL pipette tip. Next, L929 cells were exposed to different treatments and photographs were taken at different time points (0 h and 24 h). The L929 cell wound healing repair was assessed using ImageJ software.

### RNA sequence for L929 cells

H_2_O_2_-CM treated L929 cells served as control group while S@M@H NPs-CM served as experiment group. RNA sequencing was performed to detect the mRNA expression of all target genes in cells. Differentially expressed genes were screened using transcript-level quantification (*P* < 0.05, fold change > 2). GO and KEGG analyses were performed to determine the biological functions of the genes and pathways influenced by S@M@H NPs treatment.

### Intracellular ROS level detection

Intracellular ROS levels were determined using a ROS assay kit based on the fluorescent dye DCFH-DA, according to the manufacturer’s instructions. Raw 264.7 cells were inoculated into discs (15 × 10^4^ per well) and incubated overnight. After different treatment, cells were incubated with 1 mL serum-free DMEM medium containing 1 μM DCFH-DA. After incubation for 30 min at 37 °C, the cells were washed three times with DMEM to remove the excess dye. Finally, the culture was either transferred to a confocal dish to be observed under CLSM or transferred to a 96-well culture plate to be measured with a microplate reader. The excitation wavelength/emission wavelengths (Ex/Em) were set at 488/525 for the microplate reader. The ROS intensity measured by the microplate reader first normalized to the OD600, and then each group was normalized by the intensity of the control group.

### Assessment of mitochondrial dysfunction induced by H_2_O_2_

Raw 264.7 cells were seeded in confocal dishes and were treated with H_2_O_2_ and S@M@H NPs at 25 μg mL^−1^. After 24 h of treatment, cells were rinsed with fresh PBS. The mitochondrial membrane potential (ΔΨm) was measured with JC-1 fluorescent probe and observed under CLSM.

### Western blot analysis

After treatment, the cells were collected and washed three times with PBS, followed by collection in RIPA buffer containing protease and phosphatase inhibitors to prepare cell lysates. After incubation on ice for 20 min, proteins were obtained by centrifugation (12,000×*g*, 10 min) at 4 °C. Equal amounts of protein were subjected to 10% sodium dodecyl sulfate–polyacrylamide gel electrophoresis (SDS-PAGE) and transferred to polyvinyl difluoride membranes. Subsequently, nonspecific binding was blocked with 5% (w/v) bovine serum albumin (BSA) for 1 h at room temperature. The samples were immunoblotted with the following primary antibodies: Sting, Tbk1, pTbk1, p65, pp65, and glyceraldehyde 3-phosphate dehydrogenase (GAPDH) at 4 °C for 8 h. The membranes were further rinsed three times with TBST and the target proteins were detected using an e-Blot Touch Imager. GAPDH was used as a control to monitor equal protein loading.

### Immunofluorescence staining of iNOS and CD206 of macrophages

H_2_O_2_-stimulated Raw 264.7 cells were incubated with free media, H_2_O_2_ and S@M@H NPs for 48 h, respectively. Raw 264.7 cells were fixed with 4% paraformaldehyde for 10 min, followed by permeabilization with 0.1% TritonX-100. After blocking, cells were incubated with the anti-iNOS and anti-CD206 antibodies, respectively. DAPI was used to stain the nucleus. Stained macrophages were visualized by using CLSM.

### Enzyme-linked immunosorbent assay (ELISA)

The IL-6, TNF-α and IL-1β levels in the supernatant of cultured Raw 264.7 cells were detected using an ELISA Kit according to the manufacturer’s protocol.

### Assessment of in vivo healing of diabetic wounds

Animal experiments were performed according to the guidelines of the Animal Care and Use Committee of Shanghai Jiao Tong University School of Medicine. The ethical license for animal experiments was SH9H-2023-A811-1. Six-week-old female KM mice were purchased from Shanghai JieSiJie Laboratory Animal Co., Ltd. The mice were intraperitoneally injected with streptozocin (dissolved in 0.1 M ice-cold citrate buffer solution, pH = 4.5) at a dose of 50 mg/kg after fasting 12 h. Then, the mice were fed with normal food. Streptozocin was administered for 5 days, and the blood glucose levels of the mice were monitored. For surgical excisional wound, the dorsal hairs of healthy or diabetic mice were shaved. A full-thickness skin wound in 10 mm diameter was made on the back of each mouse.

### Histological evaluation

The mice were anesthetized by continuous inhalation of isoflurane in a 3% isoflurane atmosphere before surgery. First, the surgical site was disinfected with 75% ethanol and the backs of the mice were shaved. A circular full-thickness wound with a diameter of 10 mm was created using scissors. The mice were randomly divided into four groups: saline (PBS), HMUiO-MnTCPP (M@H), SOD (S@H), and SOD@HMUiO-MnTCPP NPs (S@M@H). Saline-treated mice were used as controls. Wound areas were recorded and measured on postoperative days (PODs): 1, 4, 7, and 12. The wound size was quantified using ImageJ software. Mice were euthanized on days 4 and 12, and fresh tissue sections were collected and immersed in paraformaldehyde for H&E and Masson trichrome staining.

### Immunofluorescence

Skin tissues were isolated from the wound site on day 4 and day 12, followed by the immunofluorescence staining with IL-6, α-SMA, and CD31 respectively. Nuclei were stained with 4′,6-diamidino-2-phenylindole (DAPI). The sections were observed under a positive fluorescence microscope.

### Immunohistochemistry

Immunohistochemistry was used to detect STING expression on day 12.

### Statistical analysis

All quantitative results were shown as mean ± standard deviation. All mean values represent the average of all the experimental groups analyzed. Statistical comparisons were performed using a blinded counter. The statistical significance of the differences among groups was determined using one-way analysis of variance (ANOVA). Statistical significance was set at *P < 0.05, **P < 0.01, ***P < 0.001 and ****P < 0.0001.

## Results

### Characterization of S@M@H NPs

**Fig. 1:**
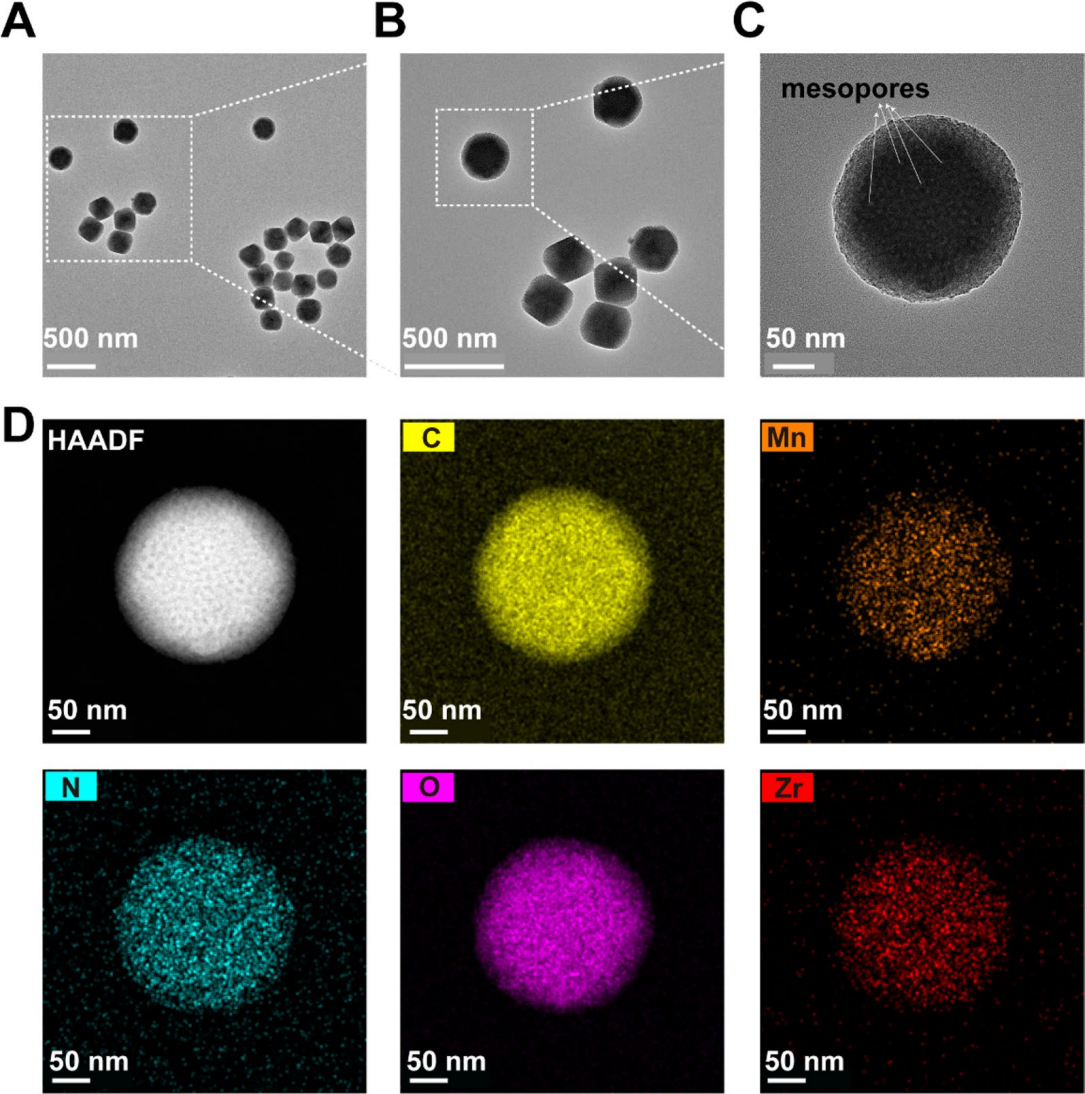
The characterization of S@M@H NPs. **A**–**C** Typical TEM images of HMUiO-MnTCPP (white arrows indicates mesopores). **D** Scanning transmission electron microscopy (STEM) image and element mapping of Mn, N, Zr, O, and C for the as-synthesized HMUiO-MnTCPP. MnTCPP Mn-meso-tetra(4-carboxyphenyl) porphine

The monodisperse spherical SOD@HMUiO-MnTCPP nanoparticles (S@M@H NPs) were obtained through refined control of the synthesis parameters. The X-ray diffraction (XRD) pattern of S@M@H NPs present characteristic peaks of the pristine UiO-66 structure, confirming the formation of a crystalline wall and maintaining the excellent crystallinity after the immobilization of SOD (Additional file 1: Fig. S1). The nitrogen sorption isotherm exhibits a combination of type I and type IV curves (Additional file 1: Fig. S2). The vertical N_2_-sorption that occurs at a low relative pressure can be ascribed to the presence of intrinsic micropores. Meanwhile, the H2 hysteresis loop between approximately 0.45–0.8 relative pressure discloses the additional presence of large mesopores. Another hysteresis loop at a relative pressure between 0.9 and 1.0 is attributed to the interparticle spaces among the NPs. The uniform mesopore diameter is determined to be about 9.6 nm as indicated in the pore-size distribution profile calculated by the Barrett–Joyner–Halenda (BJH) model using the adsorption branch (Additional file 1: Fig. S3), which could provide sufficient space for the immobilization of SOD enzymes. Transmission electron microscopy (TEM) images further confirmed that unique mesopores were distributed over the entire NPs (Fig. 1A–C). The elemental mapping results demonstrate the homogeneous distribution of Zr, C, N, O, and Mn, implying the successful integration of Mn porphyrins into the frameworks (Fig. 1D). The average particle size measured by dynamic light scattering (DLS) was 205 nm, which is slightly larger than that observed in the TEM image (Additional file 1: Fig. S4).

### ROS cascade scavenging ability of S@M@H NPs

**Fig. 2:**
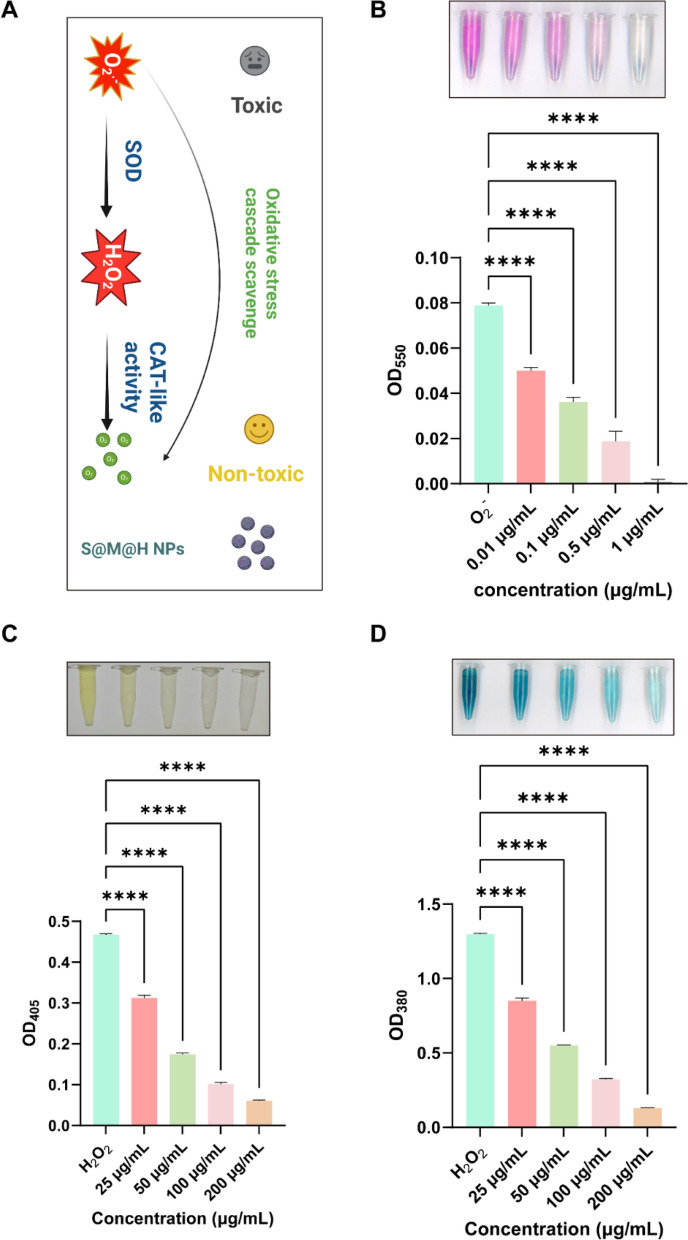
Anti-oxidant properties of S@M@H NPs in vitro. **A** Illustration of the multiple enzyme activities of S@M@H NPs, which are expected to scavenge the ROS in cells and alleviate the harsh oxidative stress microenvironment in diabetic wound. **B** O_2_^−^ scavenging ability of S@M@H NPs. **C** CAT-like activity and H_2_O_2_ depletion of S@M@H NPs determined by a catalase assay kit and TMB reagents (**D**) (****P < 0.0001)

The multiple enzyme-like activity and the excellent ROS scavenging capacity of S@M@H NPs are thus expected to alleviate the harsh oxidative stress microenvironment and act as a promising antioxidant for diabetic wound treatment (Fig. 2A). First, the SOD activity of the S@M@H NPs was evaluated. The capability of S@M@H NPs to quench O_2_^−^ was investigated using the hydroxylamine method, which could work with O_2_^−^ to generate nitrite. The latter formed a spectrophotometrically identifiable fuchsia solution at 550 nm upon the addition of a chromogenic agent. It is noteworthy that the absorbance at 550 markedly decreased with increasing S@M@H NPs concentration (Fig. 2B), indicating that S@M@H NPs possess SOD enzyme activity and eliminate O_2_^−^ in a concentration dependent manner. Next, the peroxide consumption ability of S@M@H NPs was evaluated using the ammonium molybdate method. H_2_O_2_ reacts with ammonium molybdate to form a stable yellow complex that displays an absorbance peak at 405 nm. As shown in Fig. 2C, after the addition of H_2_O_2_, the solution quickly turned yellow. With the introduction of S@M@H NPs, the corresponding absorbance of the solution gradually decreased from 0.4675 to 0.0607, indicating the catalase (CAT)-like properties of the S@M@H NPs. A TMB (3,3′,5,5′-tetramethylbenzidine) assay was performed to evaluate the H_2_O_2_ removal capacity. Ferrous ions can catalyze H_2_O_2_ to produce hydroxyl radicals, which oxidize TMB to produce a blue solution, the depth of which is proportional to the concentration of H_2_O_2_ [26]. As shown in Fig. 2D, when the S@M@H NPs concentration increased, the blue solution gradually became lighter, indicating depletion of the peroxide content. Further, the catalytic generation of O_2_ from H_2_O_2_ was tracked by a dissolved oxygen electrode. As expected, an increase in O_2_ concentrations over time could be detected when the S@M@H NPs were added to the H_2_O_2_ solution. The concentration of O_2_ was 5.405 mg L^−1^ and 10.988 mg L^−1^ respectively after incubation for 200 s and 400 s (Additional file 1: Fig. S5). Taking together, S@M@H NPs possess both SOD and CAT enzyme activities in vitro, providing feasibility for the treatment of diabetic wounds.

### S@M@H NPs alleviated STING mediated chronic inflammation in macrophages

**Fig. 3:**
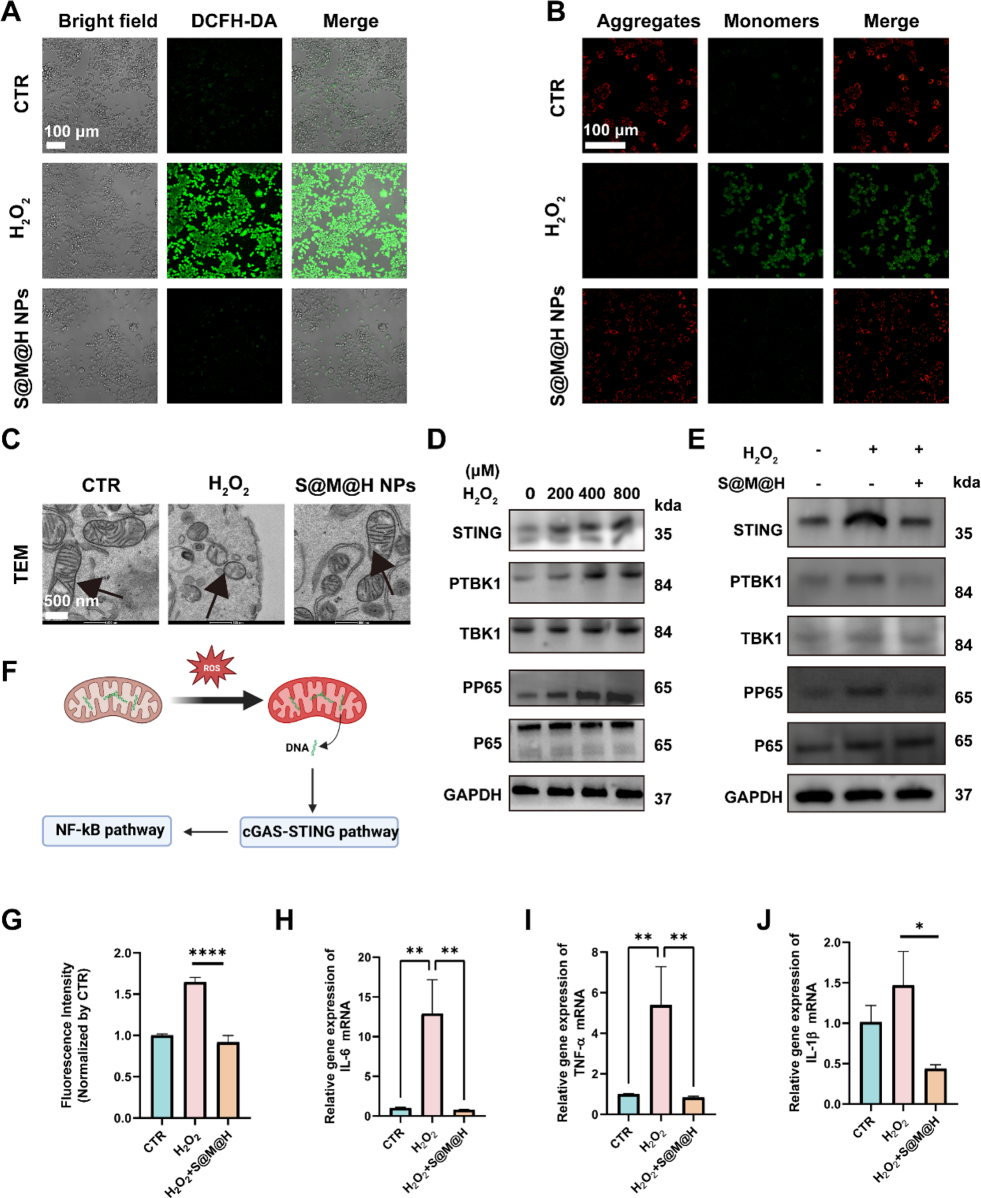
Intracellular molecular mechanism of S@M@H NPs treated Raw 264.7 cells. **A** CLSM images of Raw 264.7 cells with different treatments stained with DCFH-DA probe. **B** Mitochondrial membrane potential levels were measured with the JC-1 probe in Raw 264.7 cells following treatment with S@M@H NPs and H_2_O_2_. Red and green fluorescence, respectively, correspond to JC-1 aggregates and monomers. **C** TEM images of intracellular mitochondria in Raw 264.7 cells after H_2_O_2_ stimulation and S@M@H NPs treatment. **D** Representative Western blot analysis of STING–NF–κB pathway expression in Raw 264.7 cells treated with 0, 250, 500, 750, 1000 μM H_2_O_2_ for 4 h. **E** Representative Western blot analysis of STING–NF–κB pathway expression in Raw 264.7 cells pre-exposed 0.5 mM S@M@H NPs for 1 h followed by 250 μM H_2_O_2_ treatment for 4 h. **F** Possible mechanisms by which S@M@H NPs regulates the STING-NF-κB pathway. **G** Quantitative analysis of ROS fluorescence of Raw 264.7 cells by DCFH-DA probe. **H**–**J** The relative mRNA amount of IL-6, TNF-α and IL-1β was determined by qPCR (*P < 0.05, **P < 0.01 and ****P < 0.0001). *CLSM* confocal laser scanning microscope, *IL-6* interleukin-6, *TNF-α* tumor necrosis factor alpha, *IL-1β* interleukin-1β

Given that chronic inflammation plays a pivotal role in the delayed healing of diabetic wounds, we investigated the mechanism by which S@M@H NPs regulate macrophage-mediated chronic inflammation from the perspective of regulating oxidative stress (OS). Before exploring the potential mechanism, we evaluated the biosafety of the S@M@H NPs in macrophages, fibroblasts, and endothelial cells, which involve in the rehabilitation of wound healing [27–29]. Cell viability was evaluated using the CCK-8 assay with gradient concentrations of S@M@H NPs. The safe concentration of S@M@H NPs for macrophages, fibroblasts and endothelial cells was below 1000, 400, and 500 μg mL^−1^ respectively (Additional file 1: Figs. S6, S7, and S8). Then the DCFH-DA (2′,7′-Dichlorodihydrofluorescein diacetate) staining experiment was performed and the result indicated that intracellular ROS was upregulated in macrophages after H_2_O_2_ treatment, indicating that exogenous H_2_O_2_ causes oxidative stress in macrophages (Fig. 3A, G). Mitochondria are the main sites of ROS production, and mitochondrial dysfunction leads to increased ROS and the release of mitochondrial DNA (mtDNA) [30]. To further elucidate whether oxidative stress mediates chronic inflammation via the mitochondria, we examined mitochondrial function. JC-1 probe was used to evaluate the mitochondrial membrane potential. When the mitochondrial membrane potential is high, JC-1 accumulates in the matrix of mitochondria, forming a polymer (J-aggregates), which can produce red fluorescence. When the mitochondrial membrane potential is low, JC-1 cannot accumulate in the matrix of the mitochondria, and JC-1 is a monomer (J-monomers), which can produce green fluorescence. The decline of mitochondrial membrane potential is a landmark event of cell damage. According to the JC-1 probe, H_2_O_2_ treatment decreased the mitochondrial membrane potential of macrophages, indicating impaired mitochondrial function (Fig. 3B) [10]. Biological TEM further demonstrated that mitochondrial structures changed markedly under oxidative stress. Compared with the non-H_2_O_2_ treatment group, the mitochondrial length was significantly shorter, and the number of mitochondrial cristae was significantly reduced, indicating that S@M@H NPs can increase the size of mitochondria and the number of mitochondrial cristae to rescue unhealthy mitochondria under OS (Fig. 3C) [31]. Further mtDNA, an important source for activation of the cGAS-STING signaling pathway, has been shown to participate in many ROS related metabolic disorder diseases, such as lumbar disc degeneration, kidney fibrosis, and Alzheimer’s disease (AD) [16, 32, 33]. Therefore, we examined whether the cGAS-STING signaling pathway was activated under oxidative stress for that oxidative stress was one of the most important microenvironment characteristics in diabetic wound. Herein, we directly stimulated macrophages with H_2_O_2_ to detect whether high ROS activated the STING signaling pathway or not in macrophages. As shown in Fig. 3D, E, the STING signaling pathway was also activated in a H_2_O_2_ concentration-dependent manner, whereas S@M@H NPs effectively inhibited this activation. Figure 3F shows a potential mechanism of STING activation. When the mitochondria are subjected to oxidative stress, excessively produced ROS causes the mitochondrial DNA to oxidize. Within mitochondria, Ox-mtDNA cleaved by the endonuclease FEN1 to 500–650 bp fragments that exited mitochondria via mPTP- and VDAC-dependent channels [34]. cGAS could recognize viral, bacterial, mitochondrial, and self-DNA from dead cells to activate a cascade of signaling pathways leading to inflammation. Binding of DNA to the cGAS induces conformational changes to cGAS, which in turn catalyzes the formation of 2′,3′-cGAMP, a cyclic di-nucleotide (CDN) with a unique phosphodiester linkage. The generated cGAMP acts as a secondary messenger and activates STING and downstream proteins to initiate inflammatory response [35]. Finally, the downstream inflammatory indicators of STING, including interleukin-6 (IL-6), tumor necrosis factor alpha (TNF-α), and interleukin-1β (IL-1β) were detected and the trends were consistent with Western blot (WB) results (Fig. 3H–J). ELISA assay was further performed to measure the above inflammatory factors released from H_2_O_2_ and S@M@H NPs treated macrophages. As was shown in Additional file 1: Fig. S9–S11, H_2_O_2_ significantly increased the expression of IL-6, TNF-α and IL-1β (82.3, 319.9 and 76.7 pg mL^−1^, respectively), while the introduction of S@M@H NPs decreased the inflammatory factors significantly. Considering that the activation of p65 and the high expression of inflammatory factors are the phenotypes of M1 macrophages, we further evaluated the effect of S@M@H NPs on the polarization of macrophages. As was shown in Additional file 1: Figs. S12 and S13, after H_2_O_2_ treatment, the expression of iNOS was predominant, implying that macrophages were polarized toward M1 phenotype. In contrast, H_2_O_2_-stimulated macrophages intuitively displayed the attenuated iNOS signal and enhanced CD206 signal after incubation with S@M@H NPs. The cGAS-STING pathway is activated by DNA from various sources. Our results revealed that cGAS-STING activation in diabetic wounds might be caused by macrophage mtDNA suffering from excessive ROS. S@M@H NPs alleviated chronic inflammation in a ROS elimination way.

### Effect of S@M@H NPs on cytoprotection of L929 cells caused by inflammatory macrophages

**Fig. 4:**
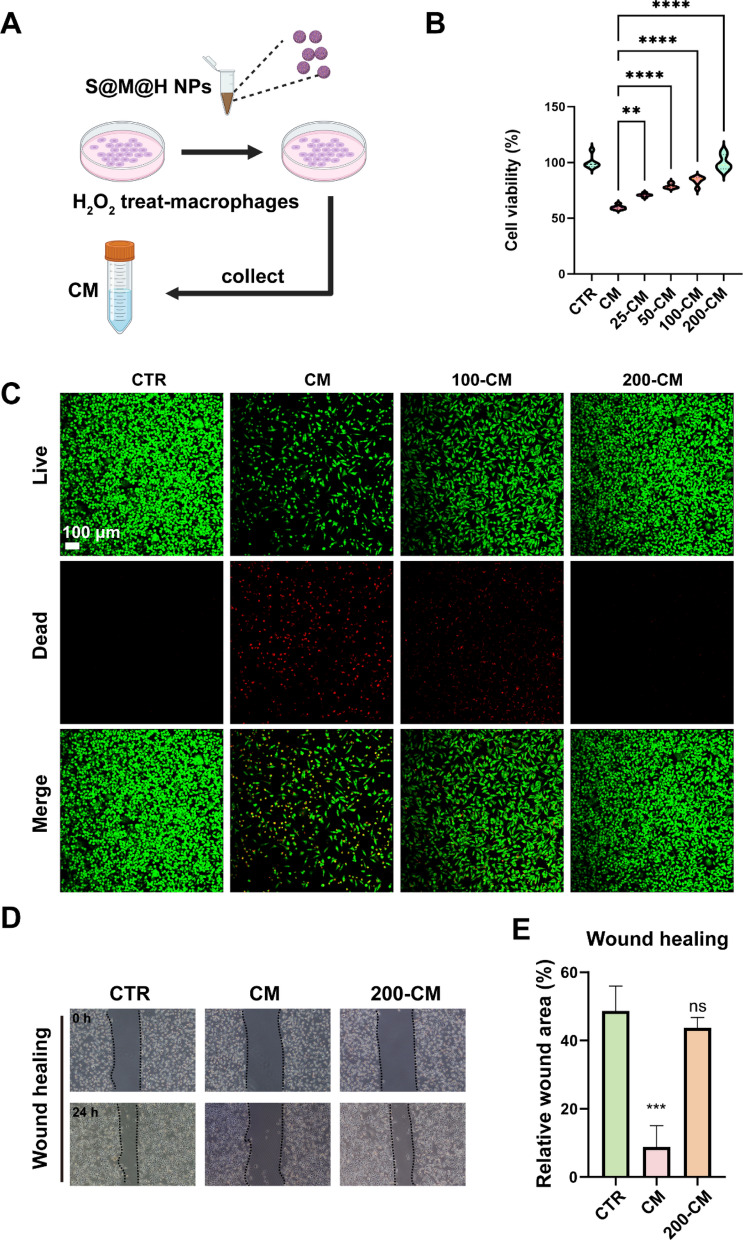
Effect of S@M@H NPs on cytoprotection of L929 cells caused by inflammatory macrophages. **A** The collection process of macrophages supernatants. **B** The survival rate of L929 cells after treatments with H_2_O_2_-CM and S@M@H NPs-CM. 25-CM, 50-CM, 100-CM, 200-CM denoted 25, 50, 100, 200 μg mL^−1^ S@M@H NPs treated macrophages supernatant. **C** Live/dead staining result of L929 cells after treatments with H_2_O_2_-CM and S@M@H NPs-CM. **D** Results of wound healing experiment in vitro. **E** The quantitative result of wound healing (**P < 0.01, ***P < 0.001 and ****P < 0.0001)

Inflammatory macrophages can interact with surrounding cells and regulate cells participating in wound healing by secreting factors [36–38]. Fibroblasts have a crucial effect on the wound healing process. In addition to supporting the wound closure process by dissolving the fibrin clot and secreting extracellular matrix, fibroblasts produce growth factors to promote the proliferation and aggregation of other epidermal cells [39]. Previous research has shown that fibroblast migration of adult diabetes mouse is inhibited, and human fibroblasts incubated under high glucose conditions likewise exhibit reduced cell migration [5]. Furthermore, diabetic wounds exhibit elevated apoptosis and reduced fibroblast proliferation [39, 40]. Whether damaged fibroblasts in diabetes are regulated by inflammatory macrophages remains to be clarified. Therefore, we explored whether STING regulation affects L929 cells through macrophages. Macrophage supernatant was used to treat fibroblasts after macrophages were pretreated with H_2_O_2_ and gradient concentrations of S@M@H NPs (Fig. 4A). We collected the supernatant, mixed it with fresh medium at a ratio of 1:1, and added it to L929 cells to observe the effect on cell function. We found that S@M@H NPs could act as effective inhibitors of STING-mediated inflammation, thus reducing the damage and cytotoxicity of H_2_O_2_-CM in L929 cells. Notably, S@M@H NPs-CM effectively attenuates inflammation-mediated damage and maintains cell viability, which is also S@M@H NPs concentration-dependent (Fig. 4B) [41]. A live/dead staining assay was performed to evaluate the protective effect of S@M@H NPs on CM-mediated damage. The addition of H_2_O_2_-CM caused fibroblasts to die, whereas the S@M@H NPs-CM effectively reduced cell death (Fig. 4C). Finally, we investigated the effect of S@M@H NPs-CM on fibroblast migration, which plays a pivotal role in wound healing. As shown in Fig. 4D, E, H_2_O_2_-CM inhibited the migration of fibroblasts, but the addition of S@M@H NPs rescued the migration ability of L929 cells. Given that impaired angiogenesis is another key factor impeding the healing of diabetic wounds, we evaluated the effects of S@M@H NPs on endothelial cells under OS [42]. Similarly, H_2_O_2_-CM significantly inhibited the migration of endothelial cells, whereas S@M@H NPs restored the migration of endothelial cells, suggesting that S@M@H NPS have the potential to promote the vascularization of diabetic wounds (Additional file 1: Figs. S14, S15).

### Transcriptome analysis of L929 cells treated with macrophage supernatants under H_2_O_2_ and S@M@H NPs conditions

**Fig. 5:**
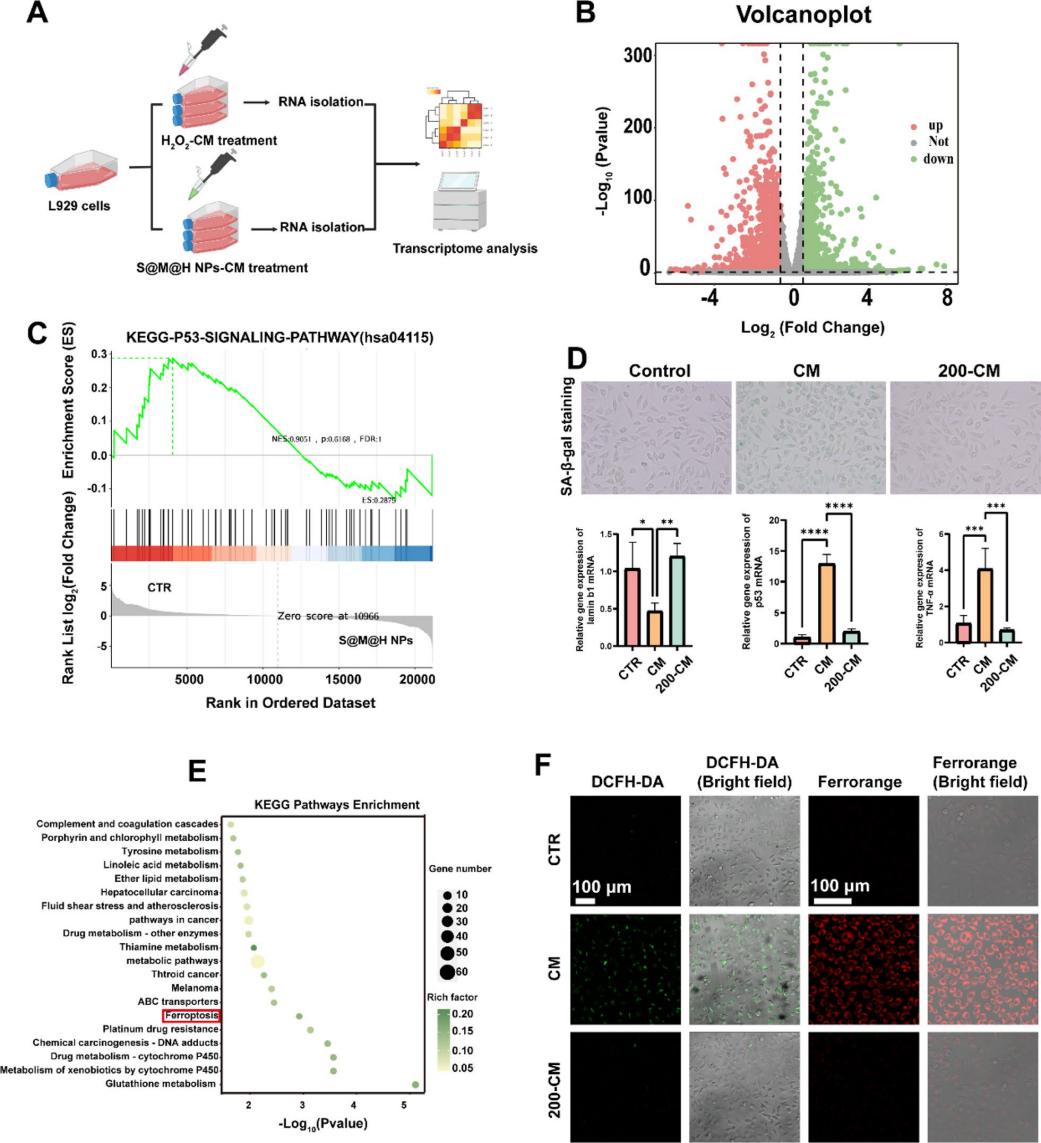
Transcriptome analysis of L929 cells treated with macrophage supernatants under NC and S@M@H NPs conditions. **A** Schematic procedure of transcriptome analysis. **B** Volcano plot of DEGs. The green dots and rose red dots represent downregulated and upregulated DEGs, respectively; the grey dots represent undifferentiated expressed genes. **C** GSEA results of downregulated DEGs. **D** Senescence-associated-β-galactosidase staining of L929 cells treated with H_2_O_2_-CM and S@M@H NPs-CM and corresponding quantitative analysis. **E** KEGG enrichment analysis of downregulated DEGs. **F** Immunofluorescence analysis of ROS (DCFH-DA) and Fe^2+^ (FerroOrange) in L929 cells stimulated with H_2_O_2_-CM and S@M@H NPs-CM for 24 h (*P < 0.05, **P < 0.01, ***P < 0.001 and ****P < 0.0001)

To further understand the molecular mechanism by which S@M@H NPs regulate fibroblasts via STING-activated macrophages, we conducted a transcriptomic analysis (Fig. 5A). As shown in Fig. 5B, the gene expression in fibroblasts significantly changed after the introduction of S@M@H NPs-CM compared to H_2_O_2_-CM. A total of 1791 genes exhibited significant changes, among which 1,099 were upregulated and 692 were downregulated (*P* < 0.05, |log_2_(fold change)| > 1). The results of gene ontology (GO) analysis, including biological processes, molecular functions, and cellular components, were shown in Additional file 1: Fig. S16. Subsequent Gene Set Enrichment Analysis (GSEA) results showed that the P53 signaling pathway played a key role in the protection of fibroblasts by S@M@H NPs (Fig. 5C). The P53 signaling pathway is a key signaling pathway involved in the regulation of cellular senescence [43]. We hypothesized that fibroblasts exhibit a senescent phenotype under H_2_O_2_-CM condition, which is the key reason for delayed wound healing [44]. Therefore, we confirmed the assumption via β-galactosidase staining. As shown in Fig. 5D, H_2_O_2_-CM induced fibroblast senescence, and S@M@H NPs protected the fibroblasts from senescence under H_2_O_2_-CM conditions. PCR results further demonstrated that S@M@H NPs effectively restored the abnormal expression of senescence-related genes, including P53, TNF-α, and Lamin b1 [43]. Cell senescence is characterized by cell cycle arrest, during which cells initiate their DNA repair program after detecting DNA damage at certain checkpoints [45]. The senescent cells in the wound bed can become the obstacle of wound healing. In fact, inflammation and aging are closely related biological events. Unresolved inflammation is characterized by the secretion of cytokines that maintain inflammation and redox stress. Mitochondrial or nuclear redox imbalance leads to DNA damage, which triggers the DNA damage response and eventually the senescent phenotype formed. Inflammation has been recognized as an endogenous factor in aging, and the elimination of inflammation could be a potential strategy for anti-aging. We believe that this is a potential explanation why inflammatory macrophages regulate fibroblast senescence. In addition, the Kyoto Encyclopedia of Genes and Genomes (KEGG) enrichment results showed that S@M@H NPs notably affected the ferroptosis signaling pathway in fibroblasts (Fig. 5E and Additional file 1: Fig. S17). This indicates that targeting ferroptosis may be an effective strategy for promoting diabetic wound healing. Ferroptosis is an iron-dependent process involved in the downstream signaling pathways of ROS generation [46]. Therefore, we conducted a preliminary examination of ferroptosis indicators. As shown in Fig. 5F, H_2_O_2_-CM significantly increased intracellular ROS levels and intracellular iron accumulation in fibroblasts. These results further confirm that OS-mediated STING activation can lead to ferroptosis. Therefore, high ROS level in diabetic wounds can delay wound healing by inducing fibroblasts senescence and ferroptosis via STING-activated macrophages.

### Healing of diabetic skin wounds in vivo treated by S@M@H NPs

**Fig. 6:**
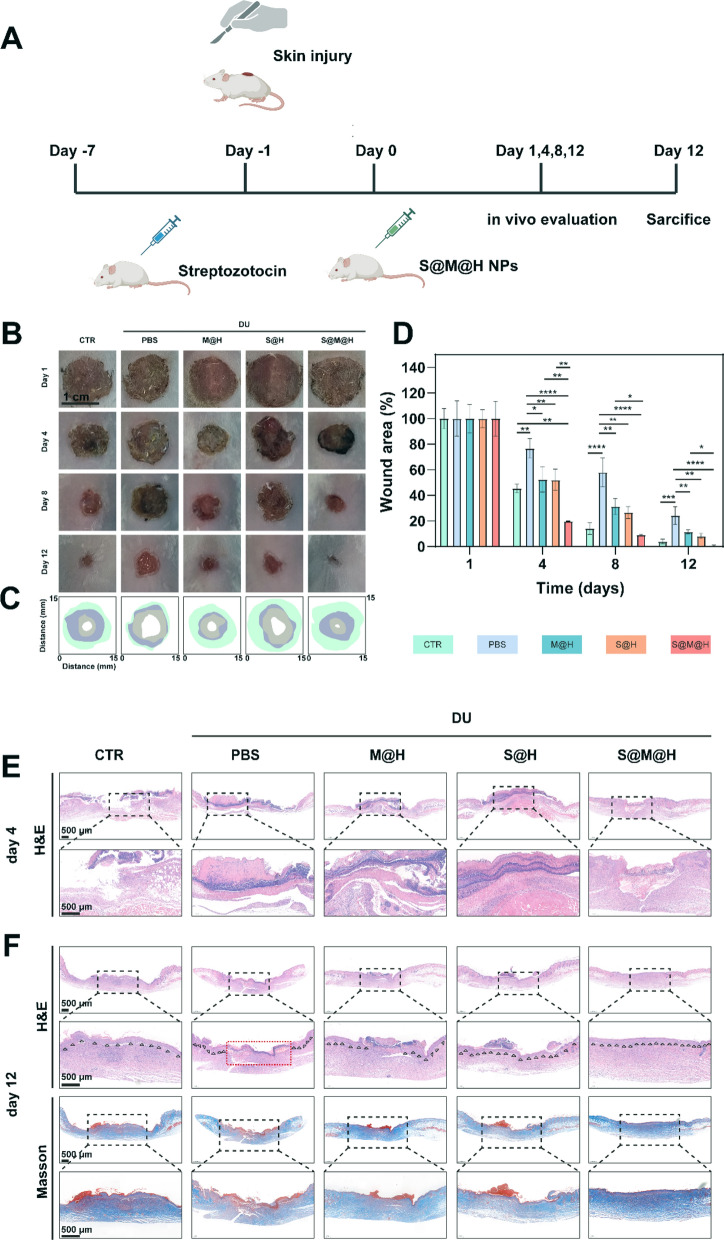
Therapeutic effect evaluation of S@M@H NPs in diabetic wounds healing. **A** Schematic diagram of the full thickness defect wound model in diabetic mice and treatment strategy. **B** The typical photographs of the wound at different days. **C** Traces of wound bed closure during 12 days. **D** Relative wound area at different time points after different treatment. **E** Histological staining of H&E in wound bed at day 4. **F** Histological staining of H&E and Masson in wound bed on day 12 (black triangle indicates re-epithelialization process, red rectangle indicates the granulation tissue) (*P < 0.05, **P < 0.01, ***P < 0.001 and ****P < 0.0001)

Prior to animal studies, we conducted a preliminary evaluation of the in vivo biosafety of S@M@H NPs. After topical administration of S@M@H NPs, the major organs of the mice, including the heart, liver, spleen, lung, and kidney, remained unchanged compared to the control groups (Additional file 1: Fig. S18). To further verify the ability of S@M@H NPs to promote wound repair in vivo, a full-thickness skin defect model in diabetic mice was established (Fig. 6A). Before the wound construction, streptozotocin (STZ) was intraperitoneally injected to establish the diabetes mellitus model. The process of wound healing on days 1, 4, 8, and 12 was recorded in Fig. 6B. Over time, the wound area of each group displayed a healing trend, while the speed differed among the groups. We conducted a quantitative analysis of the wound healing rate, and the results are shown in Fig. 6C, D. Compared to the CTR group (non-diabetic mice), the wound healing rate of diabetic mice was significantly decreased, and a large skin defect was visible on day 12. Healing of diabetic wounds was accelerated by treatment with both HMUiO-MnTCPP and SOD, which was explained by the ROS-scavenging activities of SOD and HMUiO-MnTCPP_._ Notably, the wound healing speed of the S@M@H NPs group was the fastest among the group and even better than that of the control group. On day 12, the percentages of residual wound area in each group were 3.9%, 24.1%, 11.5%, 8.1%, and 0.8%, respectively. It has been demonstrated that the spatial organization of SOD and HMUiO-MnTCPP has an enhanced ability to remove ROS. The order assembly of nature enzymes and mimic enzymes in a specific space is expected to become a novel strategy for the treatment of diabetic wounds. The excellent wound healing performance of the S@M@H NPs was further confirmed by hematoxylin and eosin (H&E) and Masson staining (Fig. 6E, F). H&E staining was performed to observe the biological mechanisms of the repair process. The results demonstrated that the wound in the S@M@H NPs group achieved re-epithelialization and granulation tissue formation during the remodeling phase, whereas wound healing was notably delayed in the control group. The change of epidermal thickness is the direct indicator of epidermis hyperplasia or differentiation. In order to investigate the effect of S@M@H NPs on the epidermis, we measured the epidermal thickness of five groups, and there seemed to be no differences among five groups (Additional file 1: Fig. S19). Collagen is an important component of skin healing and strength restoration. However, under hyperglycemic conditions, fibroblast function and collagen deposition are impaired owing to the oxidative stress microenvironment, which affects the reconstruction of granulation tissue. As shown in Fig. 6F and Additional file 1: Fig. S20, collagen deposition disorder occurred in the control group, whereas the S@M@H NPs group remained in the leading position with the highest deposition, probably because of the ROS-scavenging activity of the S@M@H NPs, which promotes fibroblast migration and protects cells from senescence and ferroptosis.

### Immunohistochemical and immunofluorescent images of wound tissue

**Fig. 7:**
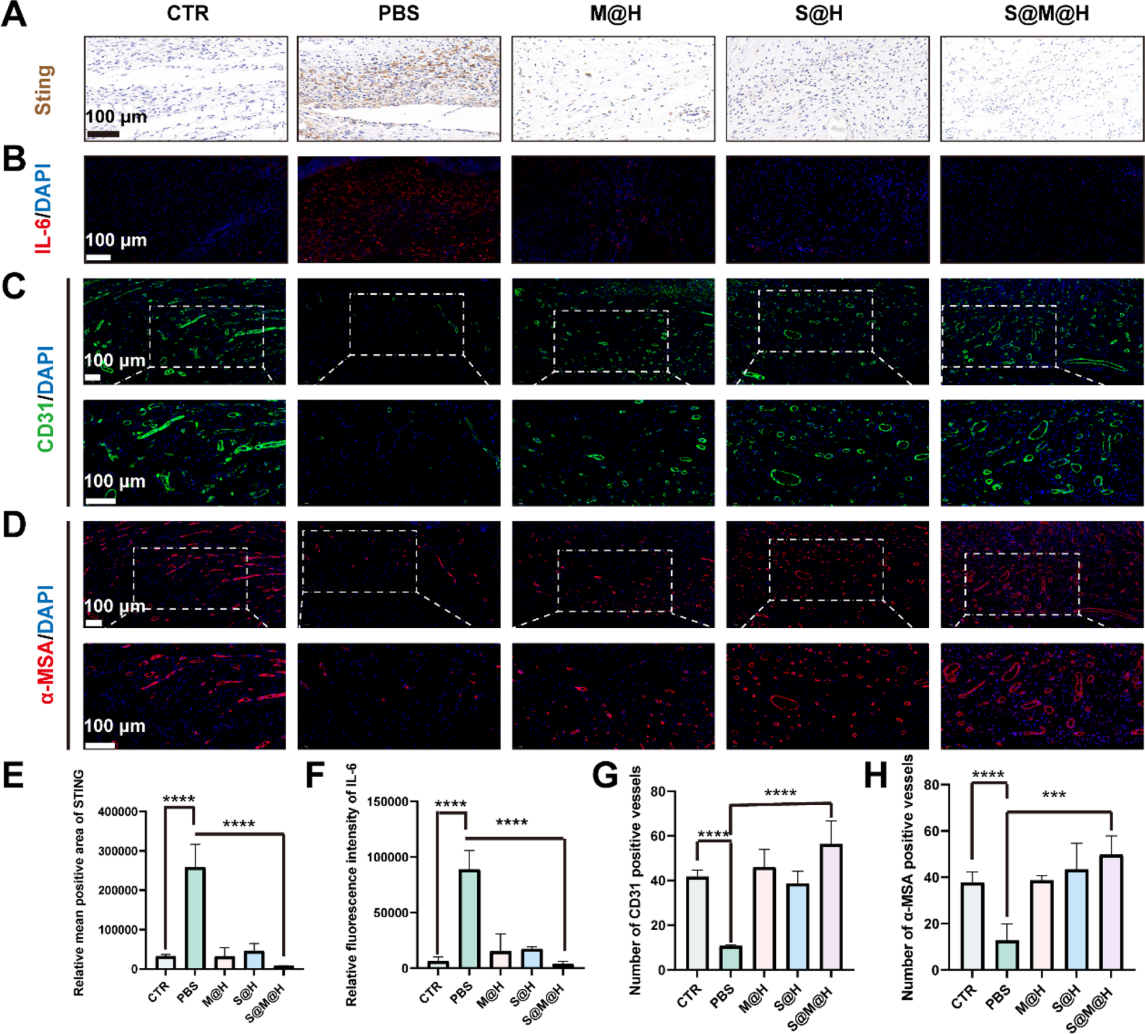
Histological evaluation of diabetic wound after S@M@H NPs treatment. **A** Immunohistochemical staining of Sting in wound bed at day 4. **B** Immunofluorescence staining of IL-6 in wound bed at day 4. Immunofluorescence staining of CD31 (**C**) and α-MSA (**D**) in wound bed at day12. **E**, **F** Relative quantitative analysis of STING and IL-6 expression. **G**, **H** Statistical data of relative expression of CD31 and α-SMA (***P < 0.001 and ****P < 0.0001). *STING* stimulator of interferon genes, *IL-6* interleukin-6

Finally, immunofluorescence and immunohistochemistry analyses were performed to further verify the mechanism by which S@M@H NPs promote diabetic wound healing. As shown in Fig. 7A, E, immunohistochemical staining showed that the PBS treated group had increased STING expression in the wound area compared to other groups. The expression of the inflammatory factor IL-6, a downstream product of STING, was also evaluated in vivo. S@M@H NPs significantly downregulated the expression of IL-6 (Fig. 7B, F). Vascular dysfunction is a key factor affecting wound healing in patients with diabetes mellitus. Considering the oxygen generation ability of S@M@H NPs, we preliminarily evaluated the effect of S@M@H NPs on vascularization in vivo by immunofluorescence staining. To detect the presence of new blood vessels, the vessel walls were stained with immunofluorescence antibodies against CD31 (a marker unique to the vascular endothelium) and α-SMA (a marker specific to vascular smooth muscle cells) [47]. As illustrated in Fig. 7C, D, G, H, obvious neovascularization was observed on the wound of normal mice, while the expression of both CD31 and α-SMA decreased in diabetic mice, indicating impaired vascularization. Wound vascularization improved after HMUiO-MnTCPP or S@M@H NPs treatment, benefiting from the CAT enzyme activity HMUiO-MnTCPP. Notably, vascularization improved after SOD treatment, which could be explained by the fact that SOD improves the function of vascular endothelial cells in diabetic wounds [48].

## Discussion

The oxidative stress microenvironment caused by the disorder of glucose and lipid metabolism plays an important role in hindering the diabetic wound healing. Excessively produced ROS promote the activation of pro-inflammatory signaling pathways, especially in macrophages, which leads to the continuous expression of inflammatory factors, including TNF-α, IL-6 and MMPS. Proteases such as superoxide dismutase (SOD), catalase (CAT) and glutathione reductase (GR) are endogenous proteins to eliminate ROS and achieve oxidative balance. However, proteases have natural limitations, including easy degradation, difficulty in preparation, and high cost. Recently, researches focusing on nanozymes to regulate oxidative stress-related diseases have made great achievement. Nanozyme is a class of non-enzymatic nanomaterials with catalytic activity, which has the advantages of simple synthesis and catalytic diversity. However, different from the high-efficiency of cascade catalysis in vivo, the catalytic efficiency of one single nanozyme is relatively low for that one nanozyme often target for a certain kind of ROS elimination. Paul successfully created an artificial protein cage housing a dual-metal-tagged guest protein that catalyzes a linear, two-step sequential cascade reaction [49]. Inspired by nature’s ingenuity, compartmentalizing ability of paracrystalline cross-β phases was utilized to colocalize sarcosine oxidase (SOX) and hemin as an artificial peroxidase in Ayan’s research [50]. For this purpose, we constructed a macroporous MOF material loaded with SOD, which also contains Mn-porphyrin ligands. Among them, SOD converts superoxide anion into H_2_O_2_ and then Mn-porphyrin further catalyzes oxygen generation via H_2_O_2_ acting as CAT. Finally, we realize efficient cascade removal of ROS by a single nanozyme system. S@M@H NPs exhibited good ROS elimination capacity both in vitro and in macrophage (Figs. 2 and 3A). Considering the excellent ROS cascade elimination ability of S@M@H NPs in vitro, we evaluated its ability to treat diabetic wounds in vivo. On day 4, the healing speed of the treatment groups was faster than that of the PBS treatment group, and we observed that the healing speed of the S@M@H NPs group was faster than that of SOD alone or HMUiO-MnTCPP alone group (Fig. 6D). It indicated that the cascade catalytic S@M@H NPs has more therapeutic potential in vivo than one single enzyme.

Then, we further studied the antioxidant mechanism of nanozymes. Differences exist in the methods of simulating cellular oxidative stress in different literatures. H_2_O_2_ was used to simulate oxidative stress according to Zhang and Cheng [51, 52]. In Geng and Chen’s study, high concentrations of glucose and LPS were used to induce cellular oxidative stress, respectively [22, 53]. In this manuscript, we introduced H_2_O_2_ to realize oxidative stress. The introduction of H_2_O_2_ significantly promoted the expression of macrophage inflammatory factors, which was consistent with the results of Chen, but the molecular mechanism was different [53]. In Chen’s work, PtAu_2_ nanoclusters activated nuclear factor erythroid 2-related factor 2 (Nrf2) expression by disrupting its association with Kelch-like ECH-associated protein 1 (Keap1), thereby upregulating the expression of endogenous anti-inflammatory and anti-oxidative products. It’s suggested that S@M@H NPs reduced the H_2_O_2_-meditated intracellular ROS of macrophages and improved mitochondrial homeostasis in our work. When mitochondria are encountered with oxidative stress, mitochondrial DNA is released from mitochondria and recognized by cGAS, thus activating cGAS-STING signaling [34]. STING-activated macrophages exhibited pro-inflammatory phenotypes. S@M@H NPs stabilized mitochondrial membrane potential and relieved mitochondrial oxidative stress by cascade clearing ROS in the microenvironment, thereby inhibiting cGAS-STING signaling activation and ROS-induced phosphorylation of p65. Cyclic cGAS-STING signaling is a classical signaling pathway by which macrophages sense and initiate inflammation and is required for the M1 polarization of macrophages [22]. We also observed that S@M@H NPs inhibited M1 polarization of macrophages induced by H_2_O_2_ via immunofluorescence (Additional file 1: Fig. S12), which was consistent with the inhibited cGAS-STING signaling. Simultaneously, H_2_O_2_ could directly destroy cellular DNA, increase the level of DNA in the cytoplasm, and then activate cGAS-STING signaling, which could be inhibited by the antioxidant *N*-acetylcysteine [16]. It’s suggested that S@M@H NPs inhibited cGAS-STING signaling via antioxidant ability.

Finally, the effect of STING-activated macrophages on fibroblasts was explored. Transcriptomic results demonstrated that the supernatant of STING-activated macrophages caused both ferroptosis and senescence of fibroblasts, which could be explained by pro-inflammatory cytokines or other secreted bioactive molecules. Ferroptosis is a nonapoptotic mode of cell death that involves iron-dependent lipid peroxidation accompanied by increased intracellular iron and ROS level. Different from other forms of cell death, ferroptosis has unique biological hallmarks, such as iron accumulation, increased lipid peroxide production, and downregulation of glutathione peroxidase 4 (GPX4) expression [54]. In this work, the supernatants of proinflammatory macrophages induced fibroblasts ferroptosis. This finding is consistent with Zhao and Zhang where inflammatory factors promote ferroptosis [55, 56]. RNA sequencing further showed that genes related to ferroptosis, including Hmox1, Tfrc and Slc7a11 were enriched. Among them, Hmox1 plays a crucial role in heme oxidation and iron metabolism. The in-depth molecular mechanism of ferroptosis in fibroblasts during the inflammatory microenvironment could be explored in future studies. Our results were also supported by the following research: according to wang, pro-inflammatory macrophages have negative regulatory effects on fibroblasts, including proliferation inhibition, spread disturbance, and collagen deposition reduction [57]. In Horiba’s research, the supernatant of M1 macrophages increased the percentages of senescence-associated β-galactosidase-positive dermal fibroblasts, whereas the supernatant of M2 macrophages decreased the percentages of senescence-associated β-galactosidase-positive dermal fibroblasts in vitro [58]. The adverse effects of inflammatory macrophages on fibroblasts were avoided after S@M@H NPs treatment. This conclusion was initially confirmed by subsequent senescence-related staining, ROS and intracellular iron detection. In summary, the S@M@H NPs promoted diabetic wound healing by inhibiting cGAS-STING pathway via ROS cascade elimination. S@M@H NPs possessed great potential for diabetic wound treatment. These findings also highlighted the potential of S@M@H NPs as a candidate for therapeutic agent not only for chronic wound healing but also for other ROS-meditated inflammatory disease, like osteoarthritis, acute kidney injury and inflammatory bowel disease [59–61]. In Wang’s study, manganese tetroxide nanozyme with both SOD and CAT like activity were successfully applied to treat osteoarthritis. More importantly, a cross-linked chondroitin sulfate hydrogel is designed as a carrier to realize the prolonged-release of nanozyme [61]. Similarly, hydrophobic Mn_3_O_4_ nanoparticles were loaded inside PTC micelles to prevent premature release during circulation in Hong’s work to treat acute kidney injury [60]. It’s implicated that the therapeutic effect of nanozymes in various diseases could be further improved by designing suitable carriers.

## Conclusion

In summary, the spatially organized bioreactors were successfully constructed and immobilized with SOD and CAT mimic enzymes, respectively. The confinement of such cascade enzymes within closely proximate nanospaces facilitated efficient mass communication of intermediate between two types of catalytic units, forming an effective biomimetic antioxidant defense system that exhibits efficient intracellular ROS-scavenging ability. More importantly, we explored the feasibility and potential mechanism of this enzyme complex in the treatment of diabetic wounds. In vitro studies demonstrated that S@M@H NPs could mitigate chronic inflammation by inhibiting the STING signaling pathway mediated by mitochondrial oxidative stress in macrophages. Additionally, the transcriptomic results suggested that S@M@H NPs rescues fibroblast senescence and ferroptosis induced by STING-activated macrophages, which was confirmed by senescence related β-galactosidase staining and iron detection. Finally, we established a diabetic mouse model with full-thickness skin defects and confirmed the therapeutic effect of S@M@H NPs on diabetic wounds in vivo. The in vivo results showed that the S@M@H NPs not only promoted wound re-epithelialization and collagen deposition, but also promoted wound vascularization.

### Supplementary Information

**Additional file 1: Figure S1.** XRD patterns of the SOD@HMUiO-MnTCPP nanoparticles (S@M@H. **Figure S2.** N_2_ sorption isotherms of S@M@H NPs. **Figure S3.** BJH pore-size distribution of S@M@H NPs. **Figure S4.** DLS results of S@M@H NPs. **Figure S5.** Evaluation of O_2_ generation from H_2_O_2_ (2.5 mM) with 200 μg mL^−1^ HMUiO-MnTCPP NPs as a catalyst. **Figure S6.** Cell viability of Raw264.7 cells after different concentrations of S@M@H NPs treatment (****P < 0.0001). **Figure S7.** Cell viability of L929 cells after different concentrations of S@M@H NPs treatment (****P < 0.0001). **Figure S8.** Cell viability of HUVEC cells after different concentrations of S@M@H NPs treatment (****P < 0.0001). **Figure S9.** ELISA analysis of IL-6 levels in the supernatant of the culture medium of Raw 264.7 cells (****P < 0.0001). **Figure S10.** ELISA analysis of TNF-α levels in the supernatant of the culture medium of Raw 264.7 cells (****P < 0.0001). **Figure S11.** ELISA analysis of IL-1β levels in the supernatant of the culture medium of Raw 264.7 cells (**P < 0.01). **Figure S12.** iNOS immunofluorescence in Raw 264.7 treated with H_2_O_2_ and S@M@H NPs. **Figure S13.** CD206 immunofluorescence in Raw 264.7 treated with H_2_O_2_ and S@M@H NPs. **Figure S14.** Protecting effect of S@M@H NPs on endothelial cell migration. **Figure S15.** Quantitative result of endothelial cell migration assay (*P < 0.05). **Figure S16.** GO analysis of differentially expressed genes. **Figure S17.** Quantitative analysis of genes related to ferroptosis. **Figure S18.** Biosafety of S@M@H NPs in vivo (photographed by leica microsystems: 92×). **Figure S19.** Quantitative analysis of the epithelium thickness. **Figure S20.** Quantitative analysis of the collagen deposition (*P < 0.05, **P < 0.01, ***P < 0.001).

## Data Availability

The data that support the findings of this study are available from the corresponding author upon reasonable request.

## Abbreviations

ROS: Reactive oxygen species
mesoMOFs: Mesoporous metal–organic frameworks
SOD: Superoxide dismutase
GR: Glutathione reductase
S@M@H NPs: SOD@HMUiO-MnTCPP nanoparticles
TEM: Transmission electron microscope
STING: Stimulator of interferon genes
MnTCPP: Mn-meso-tetra(4-carboxyphenyl) porphine
XRD: X-ray diffraction
BJH: Barrett–Joyner–Halenda
FBS: Fetal bovine serum
DLS: Dynamic light scattering
CAT: Catalase
TMB: 3,3′,5,5′-Tetramethylbenzidine
TMBE: 1,3,5-Trimethylbenzene
cGAS: Cyclic GMP-AMP synthase
OS: Oxidative stress
CCK-8: Cell Counting Kit 8
mtDNA: Mitochondrial DNA
AD: Alzheimer’s disease
IL-6: Interleukin-6
TNF-α: Tumor necrosis factor alpha
IL-1β: Interleukin-1β
WB: Western blot
CM: Macrophage supernatant
GO: Gene ontology
GSEA: Gene Set Enrichment Analysis
KEGG: Kyoto Encyclopedia of Genes and Genomes
H&E: Hematoxylin and eosin
AA: Acetic acid
BET: Brunauer–Emmett–Teller
CLSM: Confocal laser scanning microscope
BSA: Bovine serum albumin
PODs: Postoperative days
DAPI: 4′,6-Diamidino-2-phenylindole
ANOVA: One-way analysis of variance
PBS: Phosphate-buffered saline
SDS-PAGE: SDS sulfate-polyacrylamide gel electrophoresis
CTR: Control
