# Supplementary material for: Mesoporous MOFs with ROS scavenging capacity for the alleviation of inflammation through inhibiting stimulator of interferon genes to promote diabetic wound healing
Source: J Nanobiotechnology. 2024 May 13;22:246. doi: 10.1186/s12951-024-02423-6 (PMC11089722; doi:10.1186/s12951-024-02423-6)
Supplement: Supplementary file 1 — Additional file 1: Figure S1. XRD patterns of the SOD@HMUiO-MnTCPP nanoparticles (S@M@H. Figure S2. N2 sorption isotherms of S@M@H NPs. Figure S3. BJH pore-size distribution of S@M@H NPs. Figure S4. DLS results of S@M@H NPs. Figure S5. Evaluation of O2 generation from H2O2 (2.5 mM) with 200 μg mL−1 HMUiO-MnTCPP NPs as a catalyst. Figure S6. Cell viability of Raw264.7 cells after different concentrations of S@M@H NPs treatment (****P < 0.0001). Figure S7. Cell viability of L929 cells after different concentrations of S@M@H NPs treatment (****P < 0.0001). Figure S8. Cell viability of HUVEC cells after different concentrations of S@M@H NPs treatment (****P < 0.0001). Figure S9. ELISA analysis of IL-6 levels in the supernatant of the culture medium of Raw 264.7 cells (****P < 0.0001). Figure S10. ELISA analysis of TNF-α levels in the supernatant of the culture medium of Raw 264.7 cells (****P < 0.0001). Figure S11. ELISA analysis of IL-1β levels in the supernatant of the culture medium of Raw 264.7 cells (**P < 0.01). Figure S12. iNOS immunofluorescence in Raw 264.7 treated with H2O2 and S@M@H NPs. Figure S13. CD206 immunofluorescence in Raw 264.7 treated with H2O2 and S@M@H NPs. Figure S14. Protecting effect of S@M@H NPs on endothelial cell migration. Figure S15. Quantitative result of endothelial cell migration assay (*P < 0.05). Figure S16. GO analysis of differentially expressed genes. Figure S17. Quantitative analysis of genes related to ferroptosis. Figure S18. Biosafety of S@M@H NPs in vivo (photographed by leica microsystems: 92×). Figure S19. Quantitative analysis of the epithelium thickness. Figure S20. Quantitative analysis of the collagen deposition (*P < 0.05, **P < 0.01, ***P < 0.001). [file 12951_2024_2423_MOESM1_ESM.docx]

Mesoporous MOFs with ROS scavenging capacity for the alleviation of inflammation through inhibiting Stimulator of Interferon Genes to promote diabetic wound healing

Fupeng Li^1§^, Zhiyuan Mao^1§^, Yun Du^2§^, Yuehan Cui^3§^, Shengbing Yang^2^, Kai Huang^2^, Jian Yang^4^, Zhuoyuan Li^1^, Yihao Liu^2^, Jinlou Gu^4*^, Danru Wang^1*^, Chen Wang^1*^

^1^ Department of Plastic and Reconstructive Surgery,

Shanghai Ninth People's Hospital,

Shanghai Jiao Tong University School of Medicine, Shanghai, 200011, PR China

^2^ Department of Orthopaedic Surgery,

Shanghai Ninth People’s Hospital,

Shanghai Jiao Tong University School of Medicine,

Shanghai Key Laboratory of Orthopaedic Implants, Shanghai, 200011, PR China

^3^ School of Chemical Engineering，

East China University of Science and Technology,

State Key Laboratory of Chemical Engineering, Shanghai, 200237, PR China

^4^ Key Laboratory for Ultrafine Materials of Ministry of Education,

School of Materials Science and Engineering,

East China University of Science and Technology, Shanghai, 200237, PR China

* Corresponding Authors.

E-mail addresses: J. Gu (jinlougu@ecust.edu.cn),

D. Wang (wangdanru1776@163.com),

C. Wang (wangchen2369@163.com),

^§^ Fupeng Li, Zhiyuan Mao, Yun Du and Yuehan Cui contributed equally to this work.





**Figure S1.** XRD patterns of the SOD@HMUiO-MnTCPP nanoparticles (S@M@H NPs).





**Figure S2.** N_2_ sorption isotherms of S@M@H NPs.

**

**

**Figure S3.** BJH pore-size distribution of S@M@H NPs.





**Figure S4.** DLS results of S@M@H NPs.


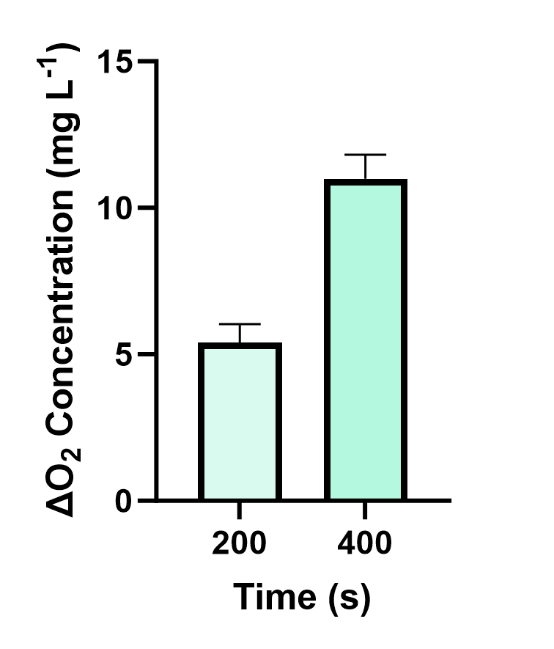


**Figure S5.** Evaluation of O_2_ generation from H_2_O_2_ (2.5 mM) with 200 μg mL^-1^ HMUiO-MnTCPP NPs as a catalyst.


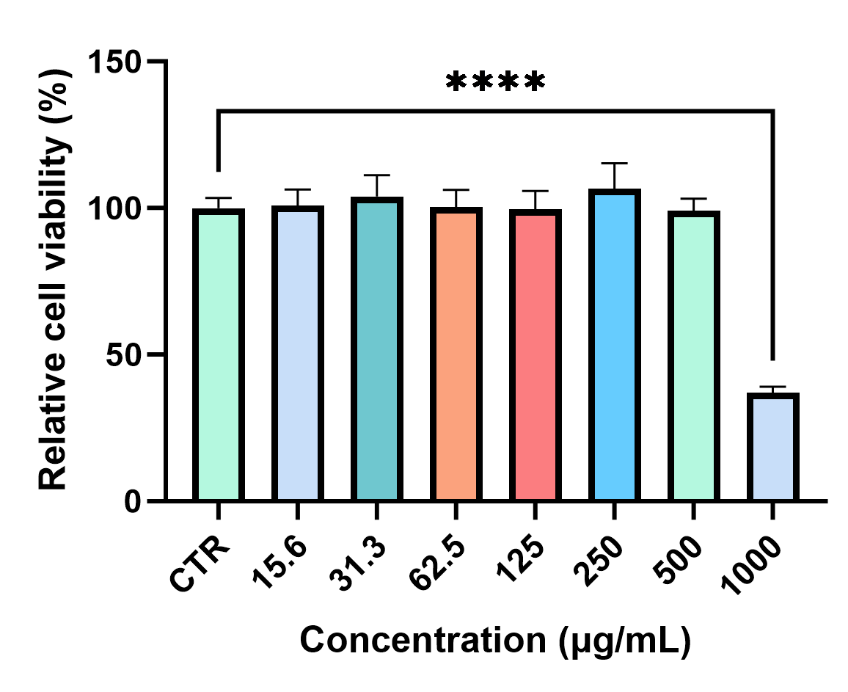


**Figure S6.** Cell viability of Raw264.7 cells after different concentrations of S@M@H NPs treatment. (*****P* < 0.0001)


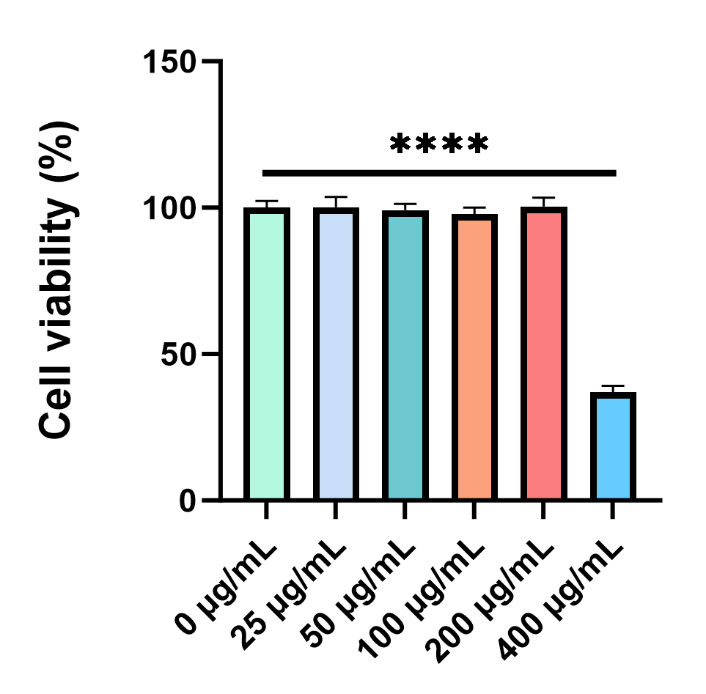


**Figure S7.** Cell viability of L929 cells after different concentrations of S@M@H NPs treatment. (*****P* < 0.0001)


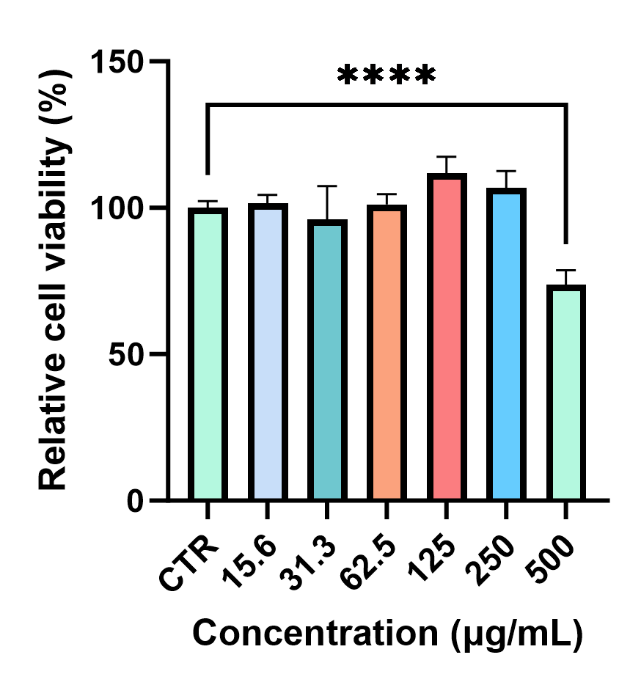


**Figure S8.** Cell viability of HUVEC cells after different concentrations of S@M@H NPs treatment. (*****P* < 0.0001)


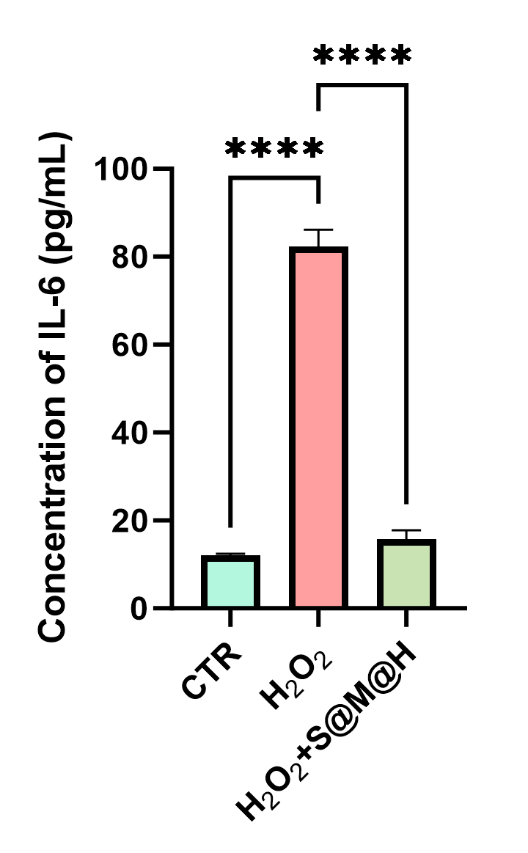


**Figure S9.** ELISA analysis of IL-6 levels in the supernatant of the culture medium of Raw 264.7 cells. (*****P* < 0.0001)


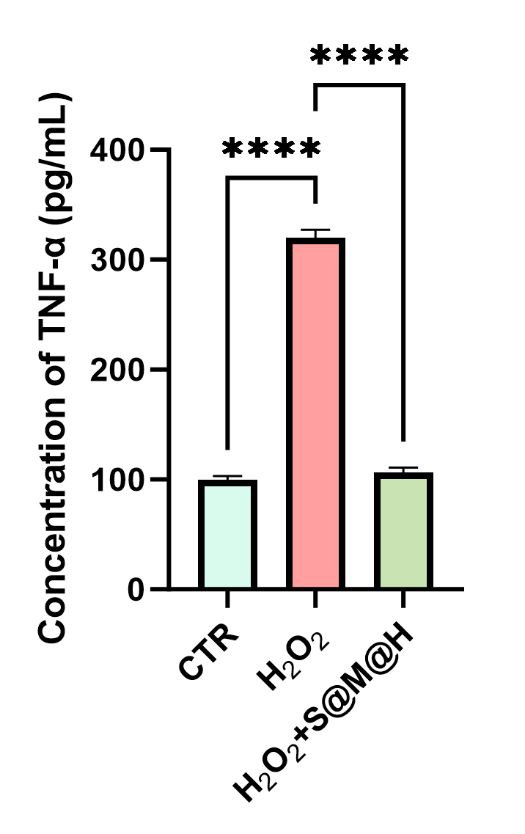


**Figure S10.** ELISA analysis of TNF-α levels in the supernatant of the culture medium of Raw 264.7 cells. (*****P* < 0.0001)


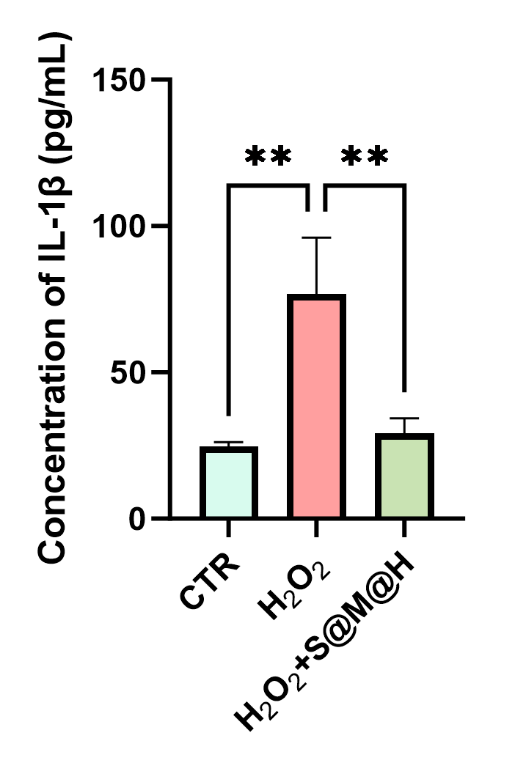


**Figure S11.** ELISA analysis of IL-1β levels in the supernatant of the culture medium of Raw 264.7 cells. (***P* < 0.01)


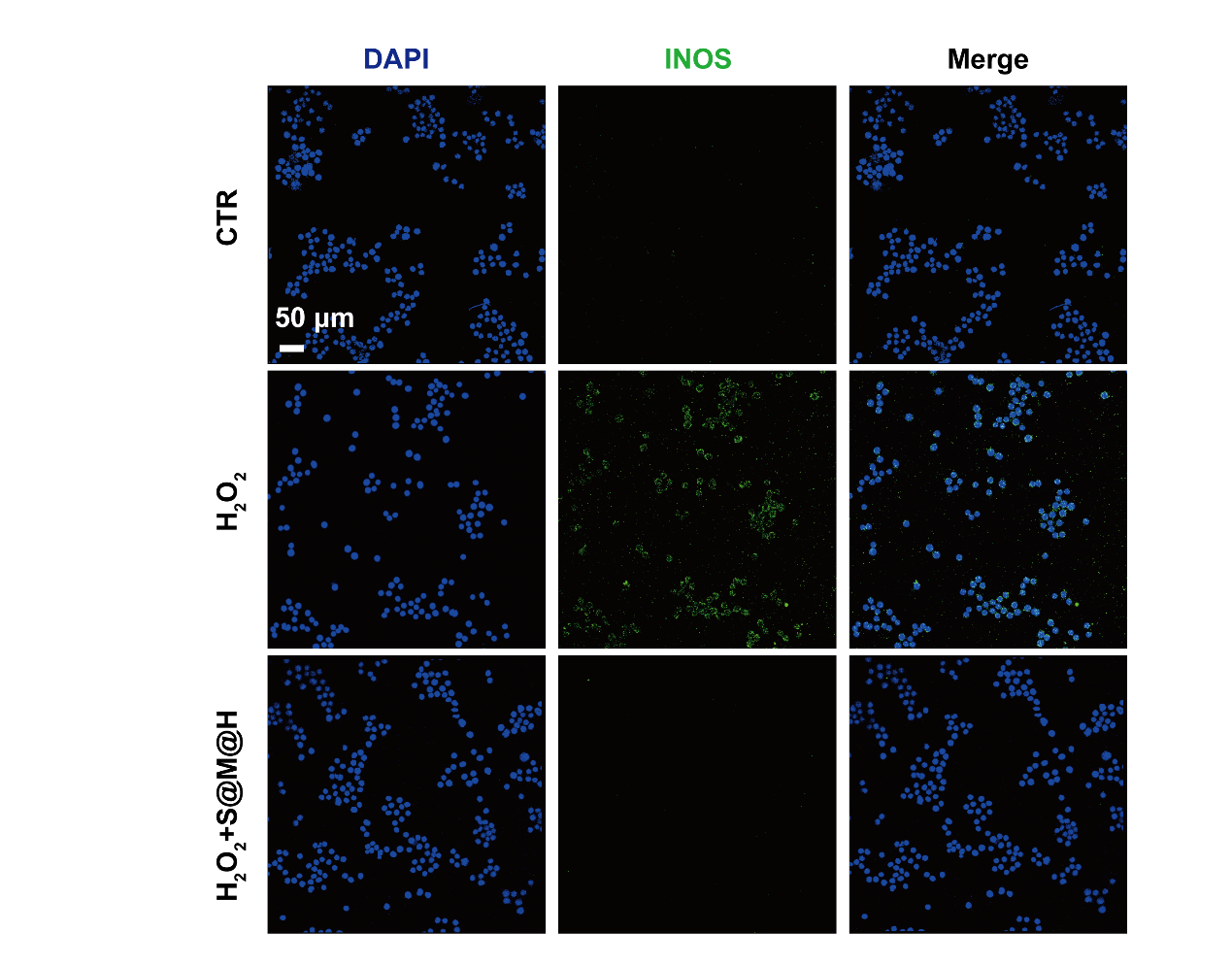


**Figure S12.** iNOS immunofluorescence in Raw 264.7 treated with H_2_O_2_ and S@M@H NPs.

z
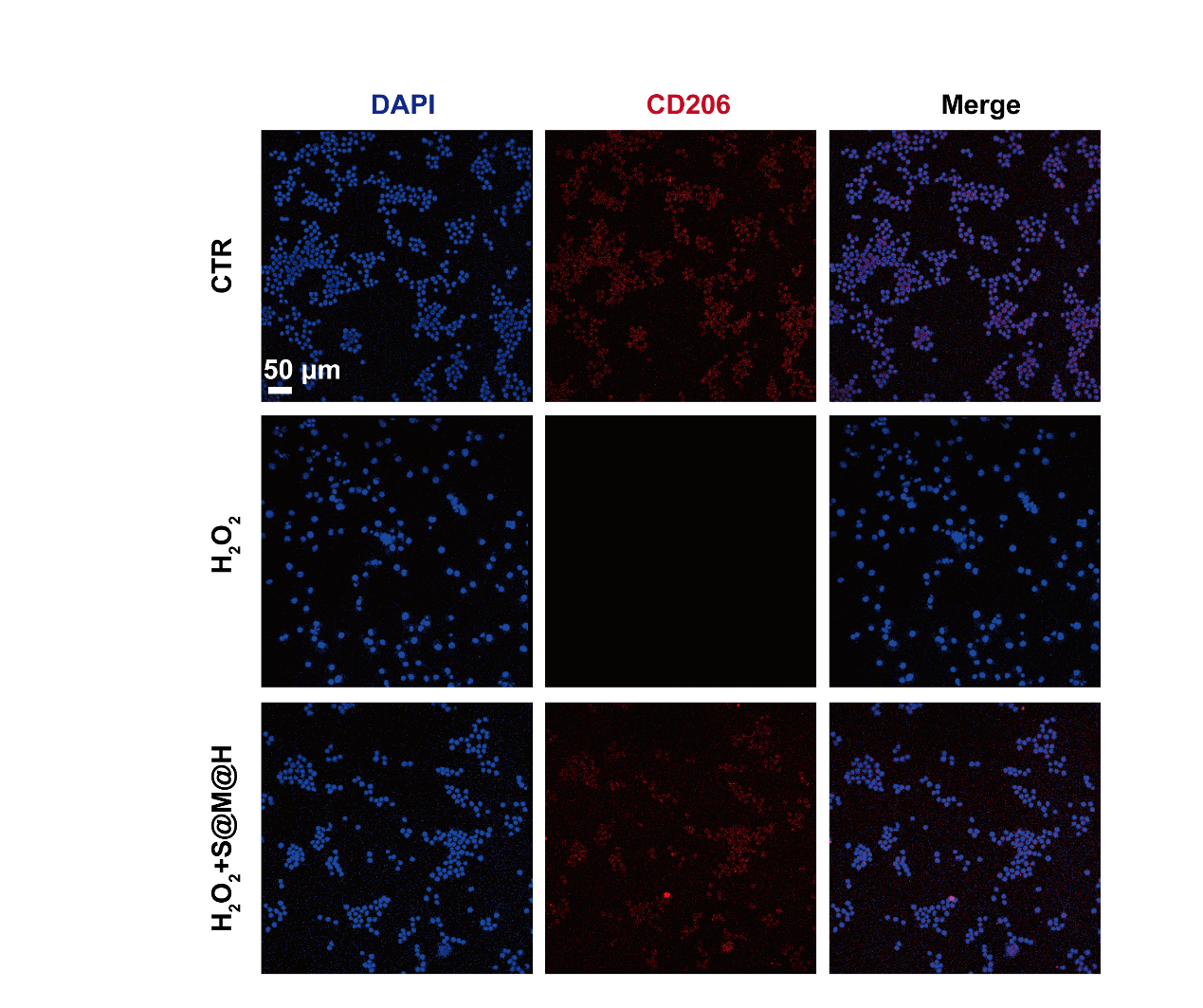


**Figure S13.** CD206 immunofluorescence in Raw 264.7 treated with H_2_O_2_ and S@M@H NPs.

**
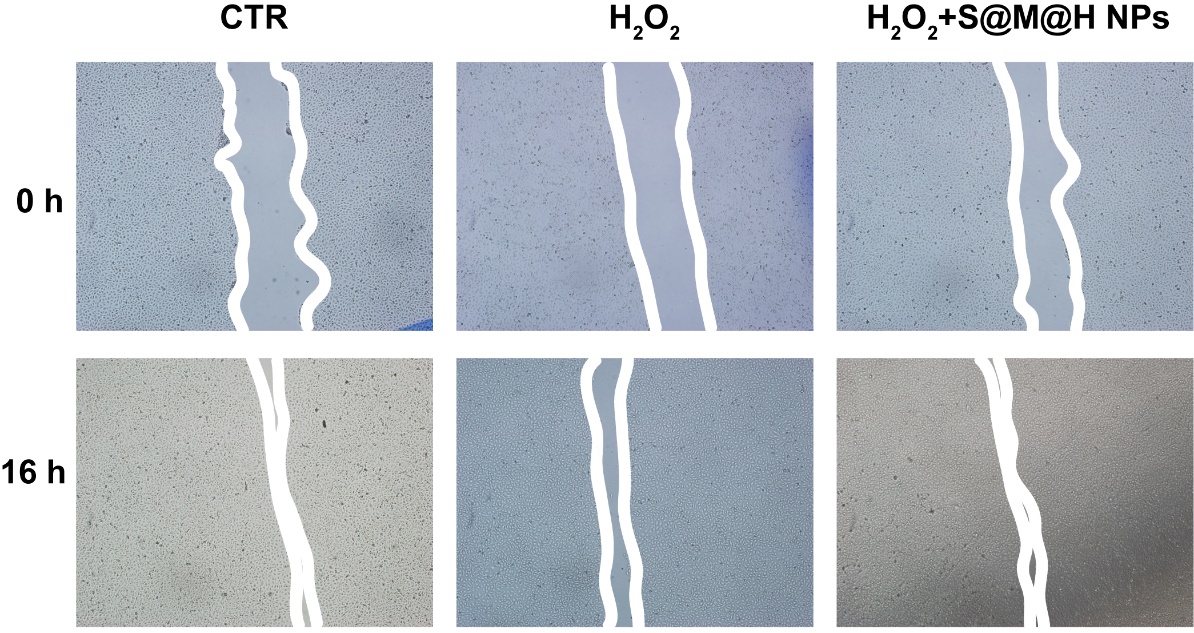
Figure S14.** Protecting effect of S@M@H NPs on endothelial cell migration.


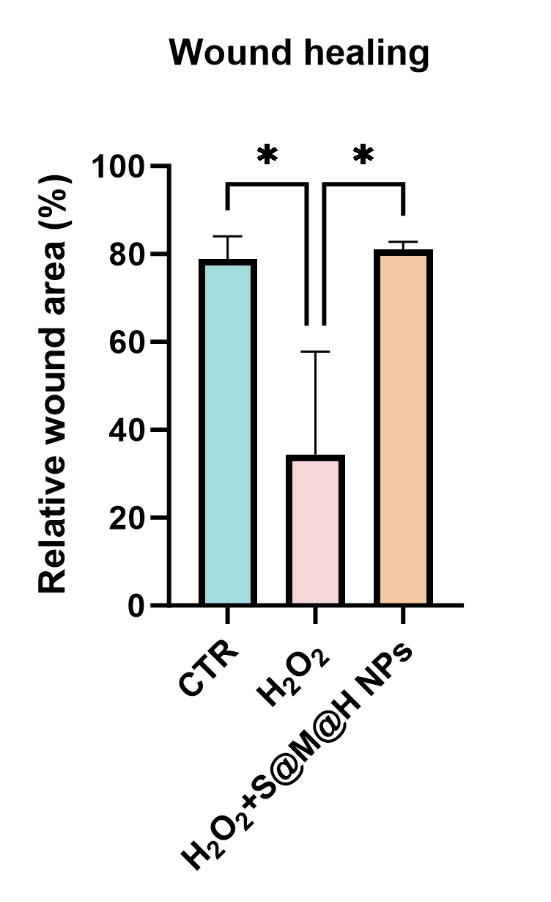


**Figure S15.** Quantitative result of endothelial cell migration assay. (**P* < 0.05)


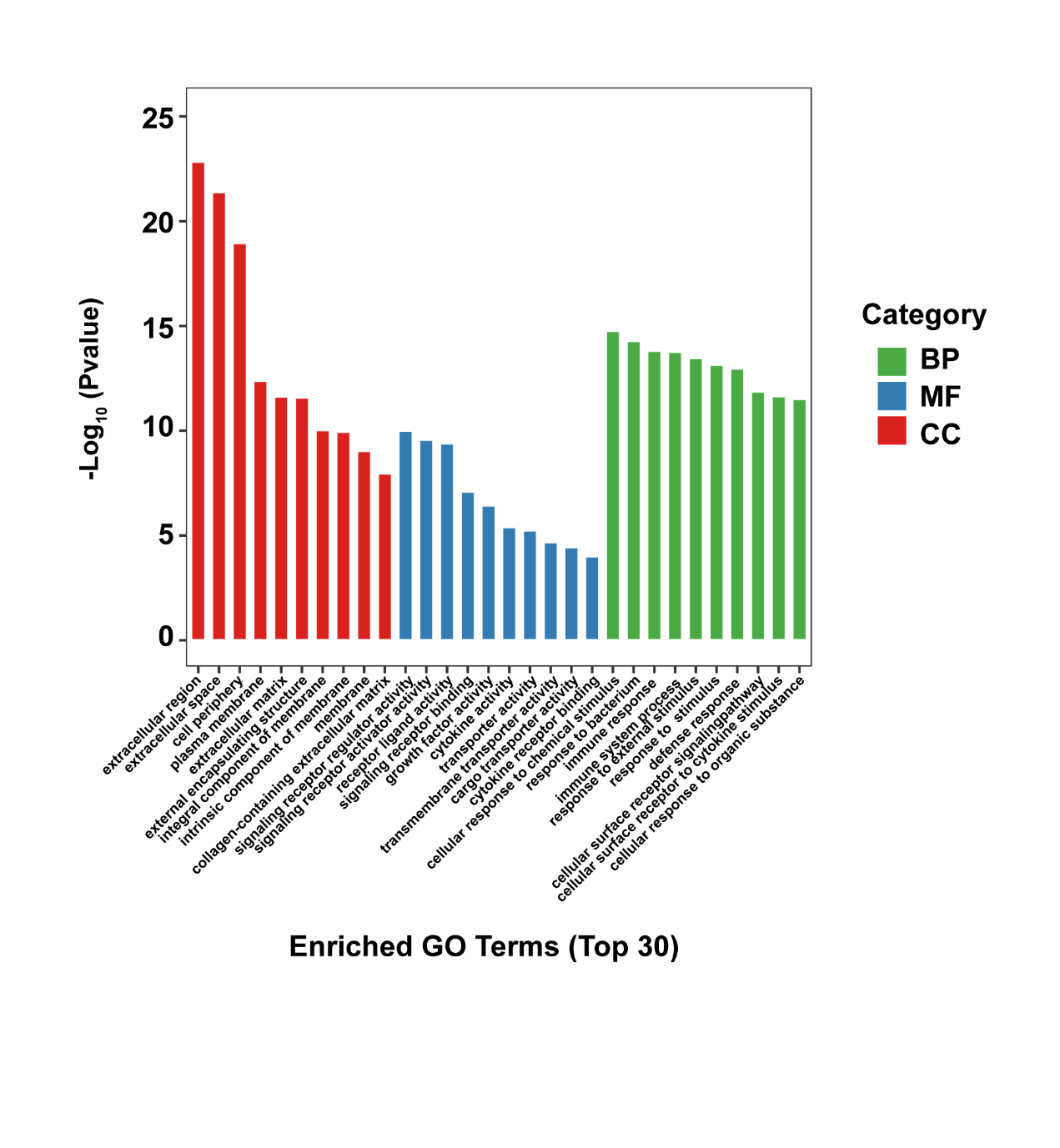


**Figure S16.** GO analysis of differentially expressed genes.

**
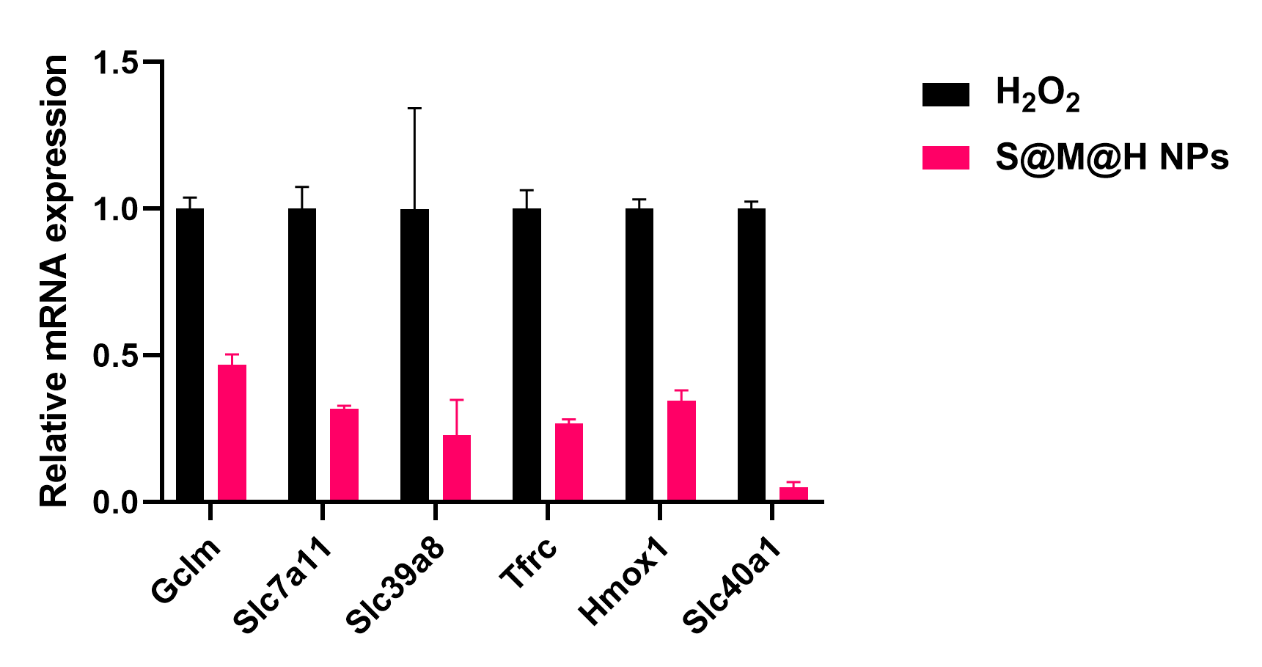
**

**Figure S17.** Quantitative analysis of genes related to ferroptosis.


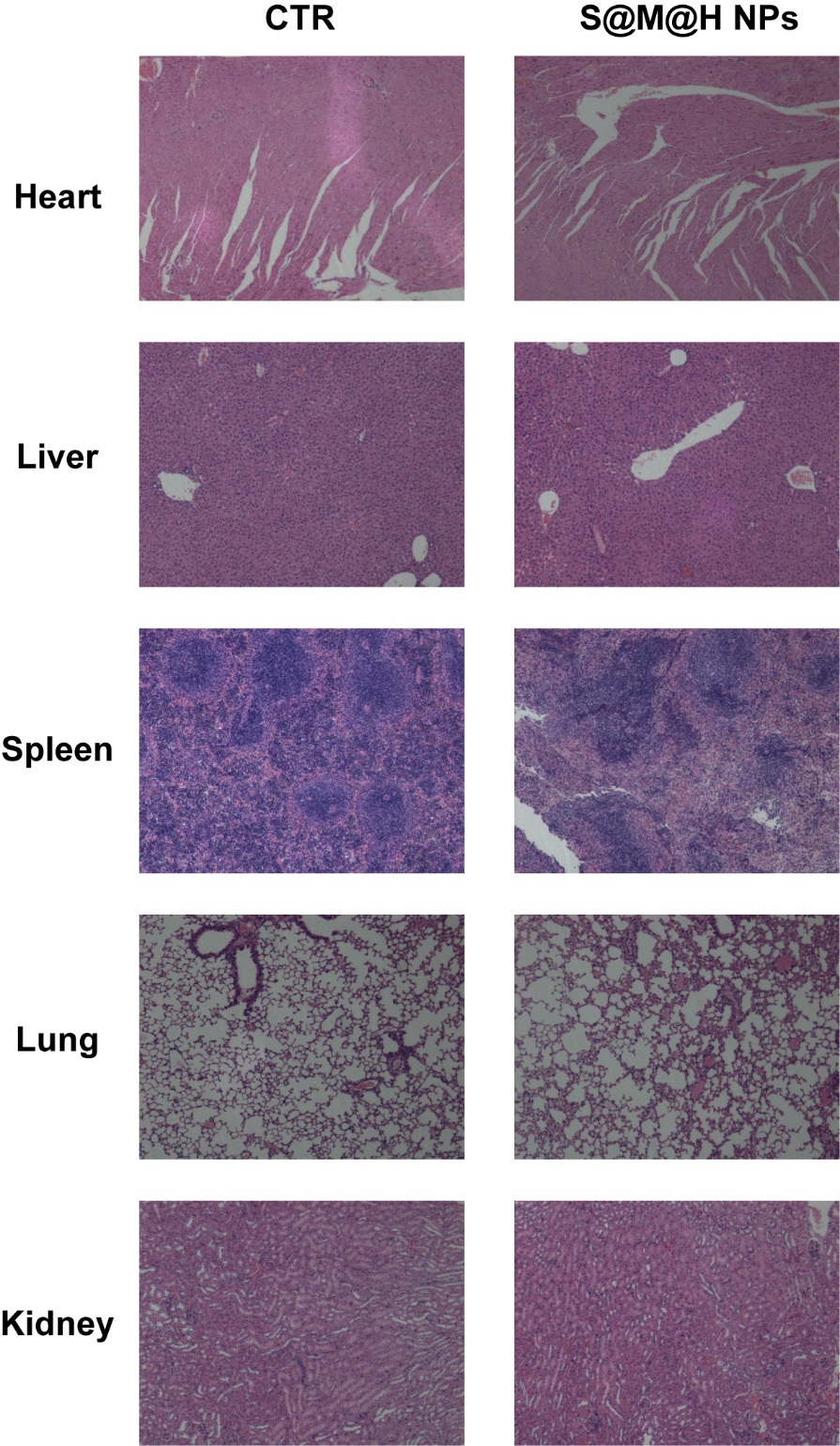


**Figure S18.** Biosafety of S@M@H NPs in vivo (photographed by leica microsystems: 92x).


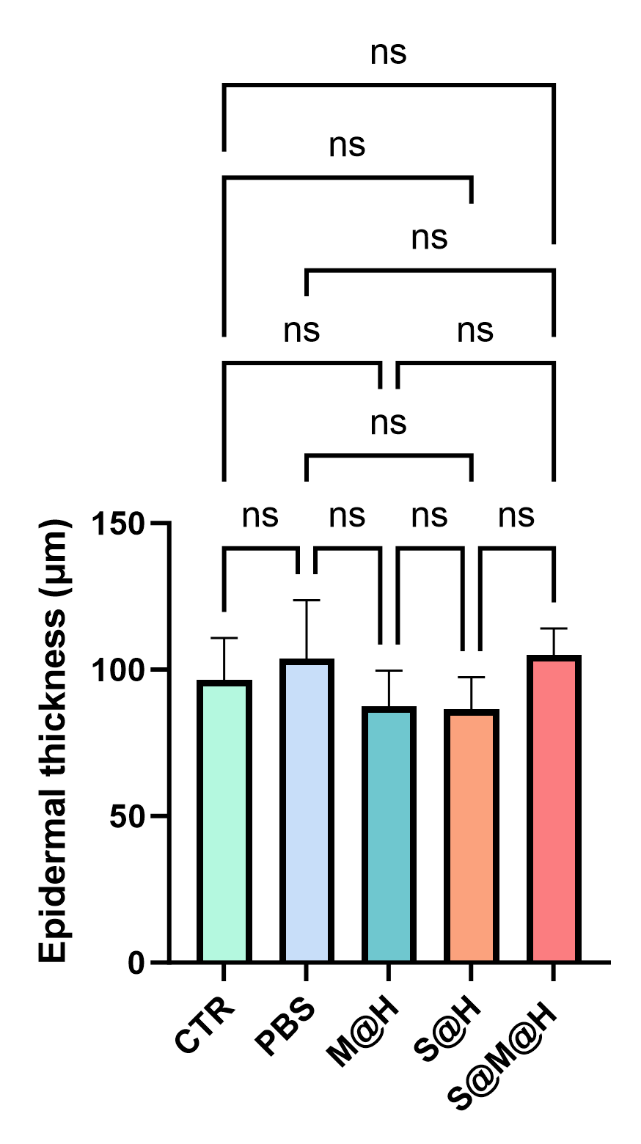


Figure S19. Quantitative analysis of the epithelium thickness.


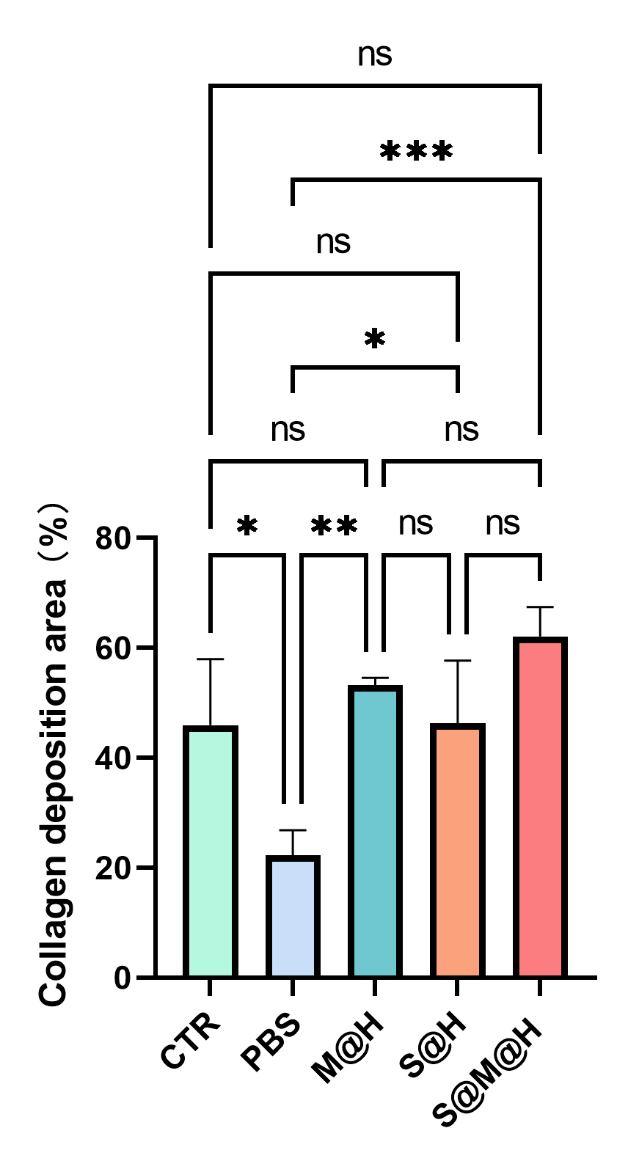


Figure S20. Quantitative analysis of the collagen deposition. (**P* < 0.05, ***P* < 0.01, ****P* < 0.001)
